# Supplementary material for: Associated Factors of Sarcopenia in Community-Dwelling Older Adults: A Systematic Review and Meta-Analysis
Source: Nutrients. 2021 Nov 27;13(12):4291. doi: 10.3390/nu13124291 (PMC8707132; doi:10.3390/nu13124291)

Table S1. Search strategy for each database.

No	Databases and access	Search terms
1	PubMed<br>(via pubmed.ncbi.nlm.nih.gov)	#1 "Aged"[MeSH Terms]<br>#2 (((Aged [Title/Abstract]) OR (older people [Title/Abstract])) OR (elderly [Title/Abstract])) OR (older adults [Title/Abstract])<br>#3 #1 OR #2<br>#4 "Sarcopenia"[MeSH Terms]<br>#5 ((((((sarcopenia [Title/Abstract]) OR (sarcopenic [Title/Abstract])) OR (muscle mass [Title/Abstract])) OR (muscle strength [Title/Abstract])) OR (muscular atrophy [Title/Abstract])) OR (gait speed [Title/Abstract])) OR (grip strength [Title/Abstract])<br>#6 #4 OR #5<br>#7 "Risk factors"[MeSH Terms]<br>#8((((risk factors [Title/Abstract]) OR (associated factors [Title/Abstract])) OR (influence factors [Title/Abstract])) OR (precipitating factors [Title/Abstract])) OR (contributing factors [Title/Abstract])<br>#9 #7 OR #8<br>#10 #3 AND #6 AND #9
2	Web of Science<br>(via webofscience.com)	#1 Aged (Topic) or older people (Topic) or older adults (Topic) or elderly (Topic)<br>#2 sarcopenia (Topic) or sarcopenic (Topic) or muscle mass (Topic) or muscle strength (Topic) or muscular atrophy (Topic) or gait speed (Topic) or grip strength (Topic)<br>#3 Risk Factors (Topic) or associated factors (Topic) or influence factors (Topic) or precipitating factors (Topic) or contributing factors (Topic)<br>#4 #1AND #2 AND #3

3	Embase<br>(via embase. com)	#1 'aged'/exp<br>#2 'sarcopenia'/exp<br>#3 'risk factor'/exp<br>#4 aged: ti,ab,kw OR elderly: ti,ab,kw OR older people: ti,ab,kw OR older adults: ti,ab,kw<br>#5 sarcopenia:ti,ab,kw OR sarcopenic:ti,ab,kw OR 'muscle mass':ti,ab,kw OR 'muscle strength':ti,ab,kw OR 'muscular atrophy':ti,ab,kw OR 'gait speed':ti,ab,kw OR 'grip strength':ti,ab,kw<br>#6 'risk factor':ti,ab,kw OR 'associated factors':ti,ab,kw OR 'influence factors':ti,ab,kw OR 'precipitating factors':ti,ab,kw OR 'contributing factors':ti,ab,kw<br>#7 #1 OR #4<br>#8 #2 OR #5<br>#9 #3AND #6<br>#10 #7 AND #8 AND #9
4	CNKI<br>(via cnki.net)	关键词:(老年人 or 老年 or 老年患者) and 关键词:(肌少症 or 肌肉减少症 or 肌肉衰减症 or 少肌症 or 骨骼肌减少症) and 题关键词:(危险因素 or 影响因素 or 相关因素)
5	CBM<br>(via sinomed.ac.cn)	#1 “老年人”[不加权:扩展]<br>#2 “危险因素”[不加权:扩展]<br>#3 “老年人”[关键词:智能] OR “老人”[关键词:智能] OR “老年患者”[关键词:智能]<br>#4 “肌少症”[关键词:智能] OR “少肌症”[关键词:智能] OR “肌肉减少症”[关键词:智能] OR “肌肉衰减症”[关键词:智能] OR “骨骼肌减少症”[关键词:智能]<br>#5 “危险因素”[关键词:智能] OR “影响因素”[关键词:智能] OR “相关因素”[关键词:智能]

		#6 (#3) OR (#1)<br>#7 (#5) OR (#2)<br>#8 (#7) AND (#6) AND (#4)
6	Wan Fang<br>(via wanfangdata.com.cn)	题名或关键词:(老年人 or 老年 or 老年患者) and 题名或关键词:(肌少症 or 肌肉减少症 or 肌肉衰减症 or 少肌症 or 骨骼肌减少症) and 题名或关键词:(危险因素 or 影响因素 or 相关因素)
7	Chinese Scientific Journals Full-Text (via cqvip.com)	题名或关键词:(老年人 or 老年 or 老年患者) and 题名或关键词:(肌少症 or 肌肉减少症 or 肌肉衰减症 or 少肌症 or 骨骼肌减少症) and 题名或关键词:(危险因素 or 影响因素 or 相关因素)

Table S2. Characteristics of the included studies.

First Author, Year	Country	Study design	Sample	Female	Age	Mean Age	Sarcopenia diagnostic criteria	Associated factors
Han 2017 [30]	China	Cross-sectional	711	50.91%	⩾60	69.52±6.55	AWGS	Diabetes, Hypertension, Hyperlipidemia
de Amorim 2019 [31]	Brazil	Cross-sectional	258	42.25%	⩾60	62.9±2.47	EWGSOP	Age, Female, Smoking
dos Santos 2015 [32]	Brazil	Cross-sectional	120	63.33%	⩾80	83.4±2.9	ASM/h^2	Male, BMI, Hypertension, Osteoporosis
Gao 2015 [33]	China	Cross-sectional	612	58.54%	⩾60	70.55±6.75	AWGS	Age, Female, Malnutrition or at risk of malnutrition
Hai 2017b [34]	China	Cross-sectional	834	50.24%	⩾60	70.63±6.62	AWGS	Smoking, Drinking
Lim 2018 [35]	Korea	Cross-sectional	3492	47.0%	⩾65	68.8±8.2	ASM/Wt	Diabetes, Hyperlipidemia
Su 2019 [23]	Japan	Cross-sectional	310	71.29%	⩾65	76.0±5.8	EWGSOP2	Age, Female, Living alone, Depression, Diabetes, Hypertension, Malnutrition and risk, Smoking, Drinking
Xu 2019a [36]	China	Cross-sectional	582	57.7%	⩾80	86.4±3.5	AWGS	Age
Yang 2020 [22]	China	Cross-sectional	483	61.90%	⩾60	66.8±4.4	EWGSOP2, EWGSOP1, AWGS, IWGS, FNIH	Age, Female, Education level, Drinking, Smoking

Meng 2014 [37]	China	Cross-sectional	771	46.56%	⩾65	73.9±6.2	EWGSOP	Female, Physical inactivity, Fall
Ishii 2014 [38]	Japan	Prospective cohort	1971	50.43%	⩾65	74.65±5.3	EWGSOP	Age
Tseng 2020 [39]	China	Cross-sectional	1025	69.76%	⩾60	71.95±6.98	AWGS	Female, Marital situation, BMI, Smoking, Drinking, Physical inactivity, Diabetes mellitus, Hypertension, Heart disease, Hyperlipidemia
Kuo 2019 [40]	China	Cross-sectional	731	47.19%	⩾65	74.9±5.35	AWGS	Age, Male, Smoking, Drinking, Abnormal Nutritional status, Osteoporosis, Hypertension, Coronary artery disease, Heart failure, Diabetes mellitus, Cancer
Akune 2014 [41]	Japan	Cross-sectional	1000	65.1%	⩾65	77.32±5.6	EWGSOP	Age, Female, Smoking, Drinking
Sousa-santos 2019 [42]	Portuguese	Cross-sectional	1500	58.13%	⩾65	74.0	EWGSOP2	Male, BMI, Education level, Drinking, Physical inactivity, Malnutrition /risk
Samper-Ternent 2016 [43]	Colombia	Cross-sectional	1442	61.03%	⩾60	70.7±7.7	EWGSOP	Age, Female, Education, Depression, ADL disability, Smoking, Drinking
Kim 2014 [44]	Japan	Prospective cohort	538	100.00%	⩾75	78.45±2.27	EWGSOP	Age, BMI, Pain, Knee pain, Falls, Osteoporosis, Heart disease, Hyperlipidemia, Knee osteoarthritis
Yu 2014 [45]	China	Prospective cohort	4000	50.0%	⩾65	72.5±5.2	EWGSOP	Age, Female, Education level, COPD, Diabetes, Hypertension, Stroke, Cancer, Smoking, Cognitive function, ADL disability
Han 2016 [46]	China	Prospective	322	61.18%	⩾60	69.76±5.44	AWGS	Age, Female, Widowed, Living alone

		cohort						
Nasimi 2019 [47]	Iran	Cross-sectional	501	49.3%	⩾65	70.3±4.6	AWGS	Age, Male, BMI, Malnutrition/risk, Smoking
Volpato 2013 [48]	Italy	Cross-sectional	483	52.17%	⩾65	83.8±5.92	EWGSOP	Age, Female, Education, BMI, Chronic liver disease
Neves 2018 [24]	Brazil	Cross-sectional	387	63.56%	⩾65	-	EWGSOP	Male, Smoking, Drinking, Fall,
Han 2015 [49]	China	Cross-sectional	1069	56.31%	⩾60	69.89±6.34	AWGS	BMI, Marital status, living alone, Drinking, Diabetes, Fall, Pulmonary disease
Dodds 2016 [50]	British	Cross-sectional	664	60.78%	⩾85	85.5±0.4	EWGSOP	Female, ADL disability, Depression, Smoking, Education years
Nakamura2020 [51]	Japan	Cross-sectional	1371	56.16%	⩾65	74.2±6.5	AWGS	Age, Female, Living alone, Hypertension, Diabetes, Hyperlipidemia, Cognitive impairment, ADL disability, Smoking, Drinking
Kurose 2020 [52]	Japan	Cross-sectional	552	68.66%	⩾60	74.6±6.7	AWGS	Age, BMI, Hypertension, Malnutrition, Anemia
Lau 2005 [53]	China	Cross-sectional	527	50.28%	⩾70	75.35±3.25	TSM/h^2	Smoking, Drinking, BMI, Physical inactivity, Diabetes mellitus, Cancer, Liver disease, Rheumatoid arthritis, Chronic obstructive airway disease
Wang 2019 [54]	China	Cross-sectional	947	50.90%	⩾60	68.78±6.25	AWGS	Age, Female, Smoking, Drinking, Physical inactivity, Malnourishment, Depression symptom
Tramontano 2017 [55]	Peruvian	Cross-sectional	222	54.05%	⩾65	73.35±6.9	IWGS	Age, Female, Physical inactivity, BMI, ADL disability, Malnourished
Figueiredo 2013 [56]	Brazil	Cross-sectional	399	0.0%	-	73.3±5.65	ASM/h^2	Age, Smoking

Moreira 2018 [57]	Brazil	Cross-sectional	680	70.33%	⩾65	76.6±6.9	EWGSOP	Female, Marital status Falls
Erkoyun 2020 [58]	Turkey	Cross-sectional	254	55.1%	⩾65	70.0	EWGSOP	Age, Female, Physical inactivity
Momoki 2016 [59]	Japan	Cross-sectional	186	100.0%	⩾65	77.7±6.8	AWGS	BMI, Living alone
Confortin 2018 [60]	Brazil	Cross-sectional	598	65.38%	⩾60	72.5±6.24	ASM/h^2	Smoking, Drinking, Physical inactivity, Cognitive decline, Depression, Falls
Badrasawi 2019 [61]	Palestine	Cross-sectional	145	51.72%	⩾60	69.5 ± 5.7	EWGSOP	Male
Wang 2020 [62]	China	Cross-sectional	515	66.21%	⩾60	70.20±6.98	EWGSOP2	BMI, Smoking, Diabetes, ADL disability
Zhang 2018 [63]	China	Cross-sectional	1148	67.94%	⩾60	74.2±7.65	AWGS	Age, BMI, Physical inactivity
Huang 2017 [64]	China	Cross-sectional	193	66.32%	⩾60	67.34±5.62	SARC-F	Diabetes
Mei 2017 [65]	China	Cross-sectional	233	69.10%	⩾60	75.78±5.79	AWGS	BMI, ADL disability
Shafiee 2020 [66]	Iran	Cross-sectional	2426	51.9%	⩾60	69.34±6.40	EWGSOP-1(Iranian), EWGSOP-2(Iranian), EWGSOP-2(European)	Age, Smoking, Education
Kim 2014b [67]	Korea	Cross-sectional	2264	58.48%	⩾65	72.72± 5.5	ASM/h^2	Chronic kidney disease, Osteopenia, Osteoporosis

Bae 2017 [25]	Korea	Cross-sectional	3901	59.7%	≥65	-	ASM/Wt	Male, Smoking, Physical inactivity, Osteoarthritis, Fall, Depression, Pain
Alexandre 2014 [68]	Brazil	Cross-sectional	1149	61.97%	≥60	71.9±0.8	EWGSOP	Female, Marital situation, Education, Smoking, Nutrition
Tyrovolas 2016 [26]	China, Ghana, India, Mexico, Russia, South Africa	Cross-sectional	18363	54.10	≥65	-	EWGSOP	Age, Education, Smoking, Drinking, Physical inactivity
Pérez-Sousa 2020 [69]	Colombia	Cross-sectional	5237	58.5%	≥60	70.4±7.8	EWGSOP2	Smoking, Drinking, Physical inactivity, Stroke, Cancer, Hypertension, Respiratory disease, Osteoporosis, Diabetes, Arthritis
Dodds 2020 [70]	British	Cross-sectional	1686	51.1%	≥69	-	SARC-F	BMI, Smoking, Drinking, Osteoarthritis, Physical inactivity
Zhang 2020 [71]	China	Prospective cohort	474	49.79%	≥59	68.11±6.18	AWGS	Age, Male, BMI, Stroke, Heart diseases, Hypertension, Diabetes, Cancer, COPD, Kidney diseases, Liver diseases
Simsek 2019 [72]	Turkey	Cross-sectional	967	60.2%	≥65	72.8±6.2	EWGSOP	Age, Physical inactivity, Diabetes, Hypertension, Malnutrition/risk
Yuenyongchaiwat 2020 [73]	Thailand	Cross-sectional	330	76.06%	≥60	66.85±5.54	AWGS	Male, Physical inactivity, Depression, Cognitive performance
Kitamura 2020 [74]	Japan	Cross-sectional	1851	50.5%	≥65	72.0±5.9	AWGS	Female, Age, Smoking, Anemia, Cognitive impairment, Depression, Physical inactivity
Xu 2019b [75]	China	Cross-	2633	57.84%	≥60	68.49±6.3	AWGS	Age, Drinking, Education level

		sectional						
Wu 2014 [76]	China	Cross-sectional	549	48.09%	⩾65	76.0±6.2	EWGSOP	Age, Male, Smoking, Drinking, Diabetes, Hypertension
Chien 2015 [77]	China	Cross-sectional	488	54.10%	⩾65	76.8 ± 6.9	ASM/h^2	Sleeping time
Domiciano 2013 [78]	Brazil	Cross-sectional	611	100.0%	⩾65	73.77±4.82	ASM/h^2	Drinking
Hu 2017 [79]	China	Cross-sectional	607	58.65%	⩾60	70.6±6.6	AWGS	Sleeping time
Landi 2012 [80]	Italy	Cross-sectional	354	66.7%	⩾80	85.8 ± 4.9	EWGSOP	Anorexia
Tsutsumimoto 2020 [81]	Japan	Cross-sectional	9496	53.0%	⩾65	74.1 ± 5.4	AWGS	Anorexia
Keng 2019 [82]	Singapore	Cross-sectional	378	47.9%	⩾60	72± 4.3	AWGS	Age, Diabetes
Souza 2019 [83]	Brazil	Cross-sectional	1078	79.22%	⩾60	74.0	EWGSOP	Female, Diabetes, Stroke, COPD, Depression
Wang 2016 [84]	China	Cross-sectional	1090	52.29%	⩾60	69.0±7.17	AWGS	Diabetes
Hsu 2014 [85]	China	Cross-sectional	335	0%	⩾65	82.95±5.35	EWGSOP	Age, Cognitive impairment, Depression
Tzeng 2020 [86]	China	Cross-sectional	1068	52.7%	⩾65	72.1	SARC-F	Physical inactivity
Daskalopoulou 2020 [87]	Cuba, Dominican Republic	Cross-sectional	7852	64.26%	⩾65	-	EWGSOP	Age, Male, Education level, Marital status, Smoking, Drinking,

	, Peru, Mexico, Puerto Rico and China							
Dutra 2015 [88]	Brazil	Cross-sectional	173	100%	⩾60	74.8±9.9	EWGSOP	Marital status, Smoking, Physical inactivity, Fall, Depression
da Silva 2016 [89]	Portuguese	Cross-sectional	253	77.9%	⩾60	100.3±2.0	ASM/h^2	Age, BMI, Female, Osteoporosis
Wu 2021 [90]	China	Cross-sectional	6172	50.26%	⩾60	68.13±6.46	AWGS	Age, Hypertension, Chronic lung diseases, Heart disease, Arthritis, Fall
Pelegrini 2018 [91]	Brazil	Cross-sectional	438	84.25%	⩾60	79.9 ± 6.0	ASM/h^2	BMI
Murphy 2013 [92]	American	Prospective cohort	2355	51.30%	⩾70	-	EWGSOP	Age, Smoking, BMI, Pain, Knee pain, Diabetes

Notes: AWGS: Asian Working Group for Sarcopenia; EWGSOP: European Working Group on Sarcopenia in Older People; ASM: appendicular skeletal mass; h: height; Wt: weight; IWGS: International Working Group on Sarcopenia; FNIH: Foundation for the National Institutes of Health; BMI: body mass index; ADL: activities of daily living; COPD: chronic obstructive pulmonary disease; TSM: total muscle mass; -: not reported.

Table S3. Subgroup analysis of the associated factors of sarcopenia with high heterogeneity.

Associated factors	Subgroup	Meta-analysis		Heterogeneity	
		Number of studies	OR (95%CI)	I^2	P
Age	Whether to adjust for confounding factors				
	Adjusted	28	1.12(1.10,1.13)	82.1	<0.001
	Unadjusted	6	1.11(1.07,1.16)	69.2	0.002
	Diagnostic criteria of sarcopenia				
	Measurement of muscle mass alone	2	1.11(0.99,1.25)	25.1	0.248
	International standards	32	1.12(1.10,1.13)	81.3	<0.001
	Geographical region				
	Asia	25	1.12(1.10,1.14)	79.1	<0.001
	South-America	5	1.07(1.01,1.13)	41.2	0.131
	Europe	1	1.22(1.14,1.30)	-	-
	North-America	1	1.12(1.09,1.16)	-	-
	Multicenter	2	1.14(1.12,1.15)	0.0	0.358
Female	Whether to adjust for confounding factors				
	Adjusted	16	1.09(0.72,1.64)	88.2	<0.001
	Unadjusted	5	1.12(0.78,1.60)	61.0	0.036
	Diagnostic criteria of sarcopenia				
	Measurement of muscle mass alone	1	5.50(1.88,16.10)	-	-
	International standards	20	1.05(0.76,1.43)	86.0	<0.001
	Geographical region				
	Asia	12	0.93(0.63,1.37)	85.9	<0.001
	South-America	6	1.56(0.65,3.77)	90.8	<0.001
	Europe	3	1.55(0.66,3.66)	76.8	0.014
Male	Whether to adjust for confounding factors				
	Adjusted	8	1.56(0.91,2.67)	87.9	<0.001
	Unadjusted	3	1.35(0.70,2.59)	69.5	0.038
	Diagnostic criteria of sarcopenia				
	Muscle mass alone	2	3.35(2.62,4.29)	0.0	0.591
	International standards	9	1.21(0.71,2.08)	85.8	<0.001
	Geographical region				
	Asia	7	1.42(0.76,2.65)	86.1	<0.001

	South-America	2	2.20(0.62,7.75)	76.4	0.04
	Europe	1	0.52(0.28,0.95)	-	-
	multicenter	1	2.82(2.22,3.58)	-	-
Overweight/obesity	Whether to adjust for confounding factors				
	Adjusted	9	0.54(0.15,1.93)	83.8	0.002
	Unadjusted	3	0.24(0.14,0.41)	88.8	<0.001
	Diagnostic criteria of sarcopenia				
	Muscle mass alone	2	0.30(0.08,1.06)	52.1	0.149
	International standards	9	0.21(0.11,0.41)	94.6	<0.001
	SARC-F	1	1.19(0.87,1.64)	40.2	0.196
	Geographical region				
	Asia	6	0.21(0.15,0.29)	34.9	0.111
	Europe	4	0.36(0.16,0.84)	92.5	<0.001
	South-America	1	0.55(0.17,1.77)	-	-
	North-America	1	1.30(1.25,1.36)	-	-
Underweight	Whether to adjust for confounding factors				
	Adjusted	13	3.78(2.52,5.66)	79.2	<0.001
	Unadjusted	1	4.00(1.00,15.97)	-	-
	Diagnostic criteria of sarcopenia				
	Measurement of muscle mass alone	2	11.8(3.25,42.86)	66.8	0.049
	International standards	12	2.75(1.95,3.87)	66.4	<0.001
	Geographical region				
	Asia	10	3.58(2.30,5.58)	81.1	<0.001
	Europe	1	4.00(1.00,15.97)	-	-
	South-America	3	4.64(2.41,8.93)	0.0	0.794
Physical inactivity	Whether to adjust for confounding factors				
	Adjusted	17	1.70(1.46,1.99)	65.5	<0.001
	Unadjusted	1	2.96(1.23,7.12)	-	-
	Diagnostic criteria of sarcopenia				
	Measurement of muscle mass alone	3	1.40(1.04,1.88)	46.1	0.098
	International standards	13	1.63(1.40,1.90)	49.3	0.013
	SARC-F	2	3.17(1.29,7.74)	88.8	0.003
	Geographical region				
	Asia	11	1.78(1.41,2.25)	64.7	<0.001
	Europe	2	1.99(1.53,2.59)	0.0	0.594

	South-America	4	2.15(1.32,3.50)	60.9	0.037
	Multicenter	1	1.25(1.08,1.45)	15.5	0.277
Marital status	Whether to adjust for confounding factors				
	Adjusted	5	1.45(0.99,2.11)	70.8	0.001
	Unadjusted	2	2.42(0.53,11.12)	70.8	0.064
	Geographical region				
	Asia	3	1.85(0.91,3.76)	54.3	0.087
	South-America	3	2.09(1.22,3.58)	48.4	0.101
	Multicenter	1	0.82(0.63,1.09)	29.3	0.234
Education level	Whether to adjust for confounding factors				
	Adjusted	9	0.95(0.92,0.98)	63.6	<0.001
	Unadjusted	2	0.81(0.59,1.12)	34.5	0.217
	Geographical region				
	Asia	4	0.95(0.93,0.98)	53.3	0.022
	Europe	3	0.82(0.72,0.93)	0.0	0.421
	South-America	2	1.02(0.99,1.06)	0.0	0.327
	Multicenter	2	0.77(0.67,0.89)	0.0	0.654
Diabetes	Whether to adjust for confounding factors				
	Adjusted	15	1.53(1.21,1.94)	64.9	<0.001
	Unadjusted	4	1.14(0.99,1.32)	0.0	0.964
	Diagnostic criteria of sarcopenia				
	Measurement of muscle mass alone	2	1.13(0.74,1.73)	0.0	0.550
	International standards	16	1.48(1.22,1.80)	62.5	<0.001
	SARC-F	1	1.09(0.79,1.50)	-	-
	Geographical region				
	Asia	16	1.38(1.12,1.71)	51.0	0.005
	South-America	2	1.96(0.83,4.67)	87.7	0.004
	North-America	1	1.12(0.94,1.33)	-	-
ADL disability	Whether to adjust for confounding factors				
	Adjusted	6	1.52(1.13,2.05)	87.8	<0.001
	Unadjusted	1	1.40(0.96,2.05)	0.0	0.884
	Geographical region				
	Asia	4	2.77(1.68,4.57)	57.7	0.069
	South-America	2	0.98(0.91,1.05)	24.1	0.268
	Europe	1	1.40(0.96,2.05)	0.0	0.884
Hypertension	Whether to adjust for confounding factors				
	Adjusted	9	1.02(0.83,1.25)	65.5	0.003
	Unadjusted	4	0.90(0.73,1.11)	0.0	0.826
	Diagnostic criteria of sarcopenia				
	Measurement of	1	0.64(0.27,1.52)	-	-

	muscle mass alone				
	International standards	12	0.99(0.85,1.16)	53.4	0.009
	Geographical region				
	Asia	11	0.94(0.76,1.16)	57.0	0.006
	South-America	2	1.00(0.66,1.52)	34.0	0.218
Cognitive impairment	Whether to adjust for confounding factors				
	Adjusted	5	1.78(1.12,2.84)	64.2	0.007
	Unadjusted	1	0.98(0.65,1.49)	-	-
	Diagnostic criteria of sarcopenia				
	Measurement of muscle mass alone	1	1.54(0.31,7.55)	67.7	0.078
	International standards	5	1.66(1.03,2.67)	74.1	0.001
	Geographical region				
	Asia	5	1.66(1.03,2.67)	74.1	0.001
	South-America	1	1.54(0.31,7.55)	67.7	0.078
Stroke	Geographical region				
	Asia	2	2.92(1.60,5.31)	0.0	0.353
	South-America	2	0.78(0.57,1.05)	0.0	0.397
Osteopenia/ osteoporosis	Whether to adjust for confounding factors				
	Adjusted	4	1.97(1.15,3.38)	53.5	0.057
	Unadjusted	2	3.73(1.53,9.09)	77.2	0.004
	Diagnostic criteria of sarcopenia				
	Measurement of muscle mass alone	3	7.30(2.72,19.57)	52.2	0.099
	International standards	3	1.76(1.19,2.62)	53.5	0.056
	Geographical region				
	Asia	3	2.41(1.49,3.90)	39.8	0.126
	South-America	2	1.64(0.79,3.40)	59.7	0.115
	Europe	1	16.99(5.52,52.29)	-	-
Depression	Whether to adjust for confounding factors				
	Adjusted	8	1.58(1.21,2.06)	77.2	<0.001
	Unadjusted	3	1.14(0.78,1.69)	0.0	0.606
	Diagnostic criteria of sarcopenia				
	Measurement of muscle mass alone	2	1.26(0.97,1.64)	0.0	0.931
	International	9	1.52(1.15,2.01)	75.0	<0.001

	standards				
	Geographical region				
	Asia	6	1.69(1.31,2.19)	48.0	0.062
	South-America	4	1.03(0.98,1.08)	0.0	0.686
	Europe	1	1.25(0.81,1.94)	0.0	0.805

Notes: OR: odds ratio; -: none.

Figure S1. Forest plot of the association between age and sarcopenia.

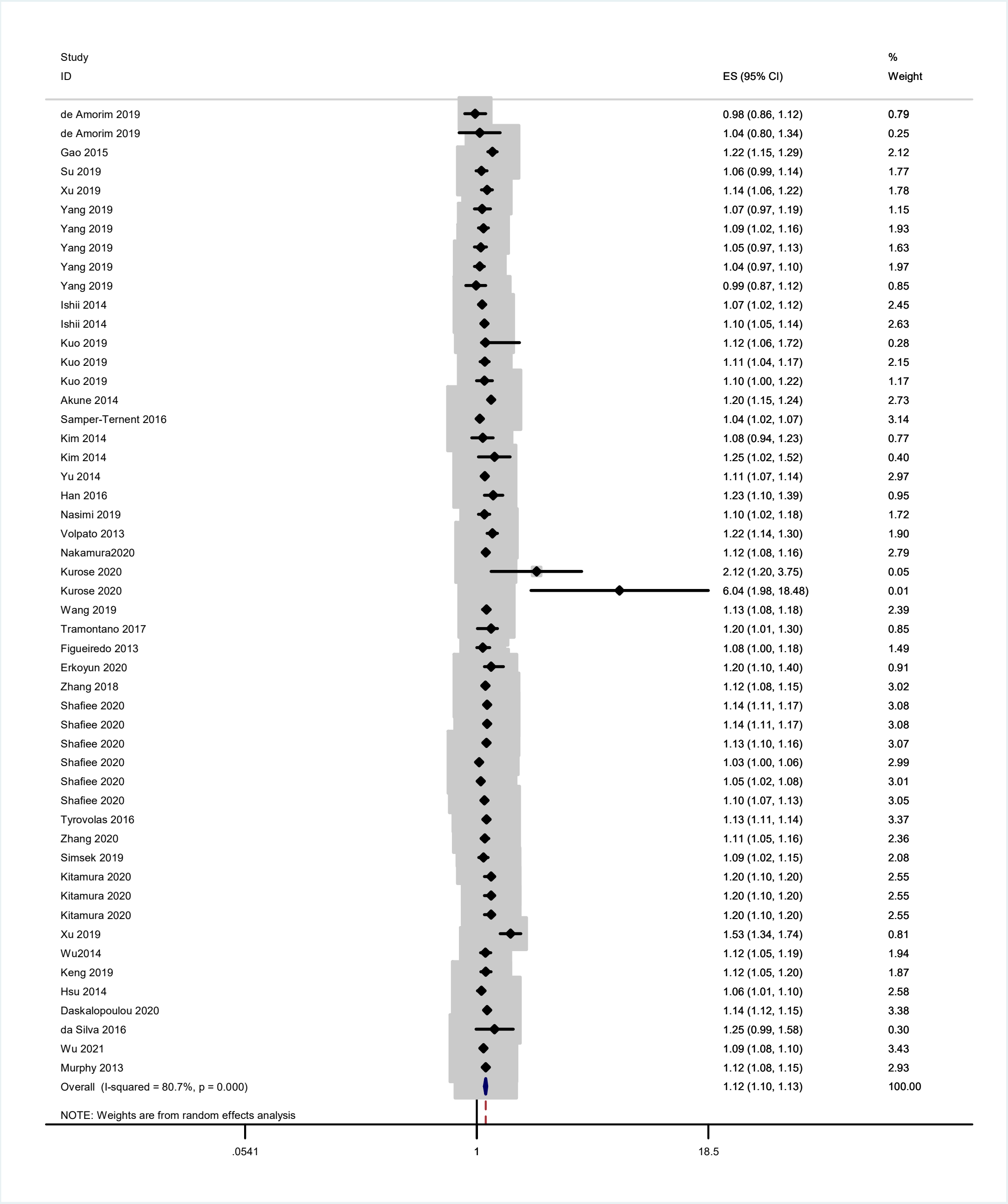

Figure S2. Forest plot of the association between marital status and sarcopenia.

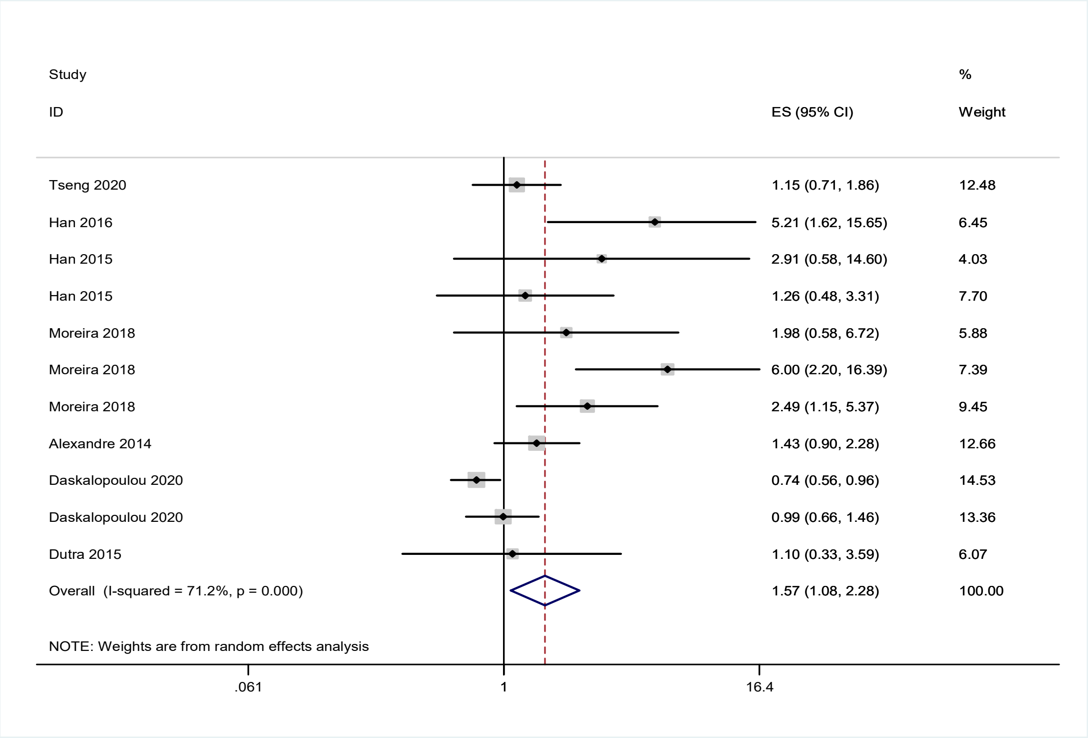

Figure S3. Forest plot of the association between low BMI (underweight) and sarcopenia.

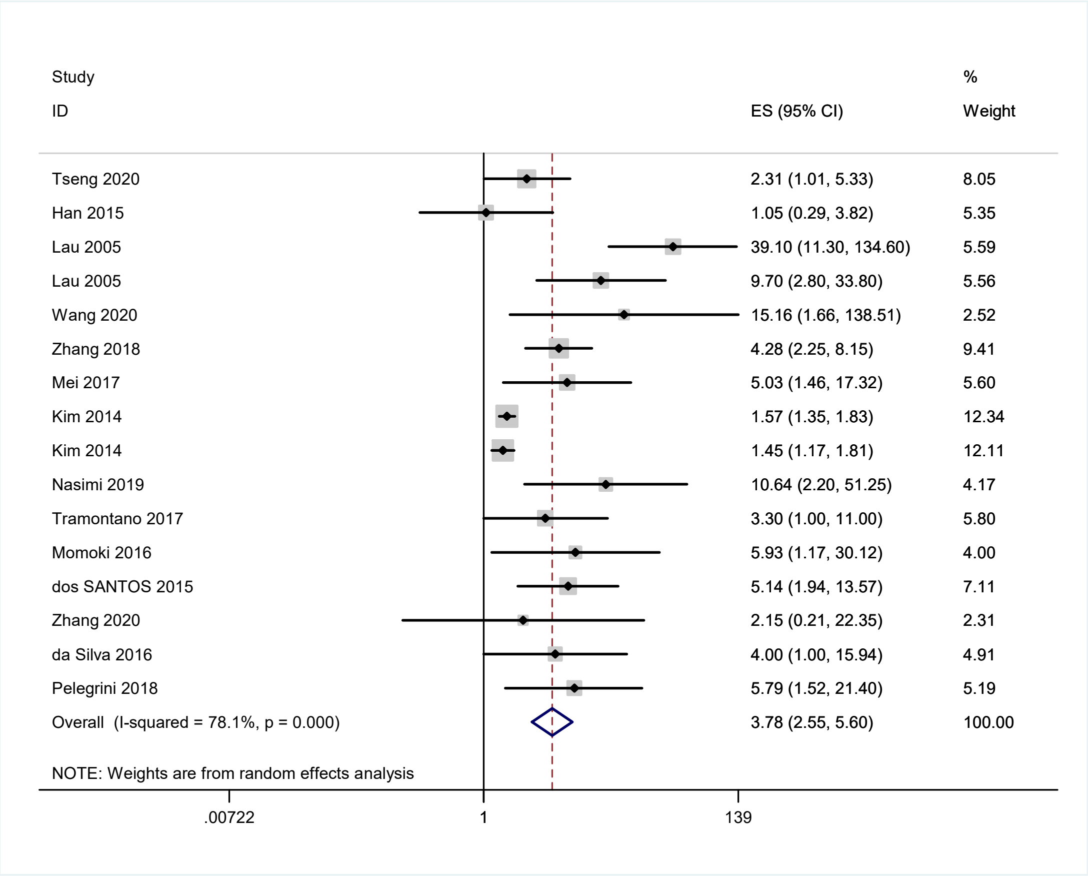

Figure S4. Forest plot of the association between ADL disability and sarcopenia.

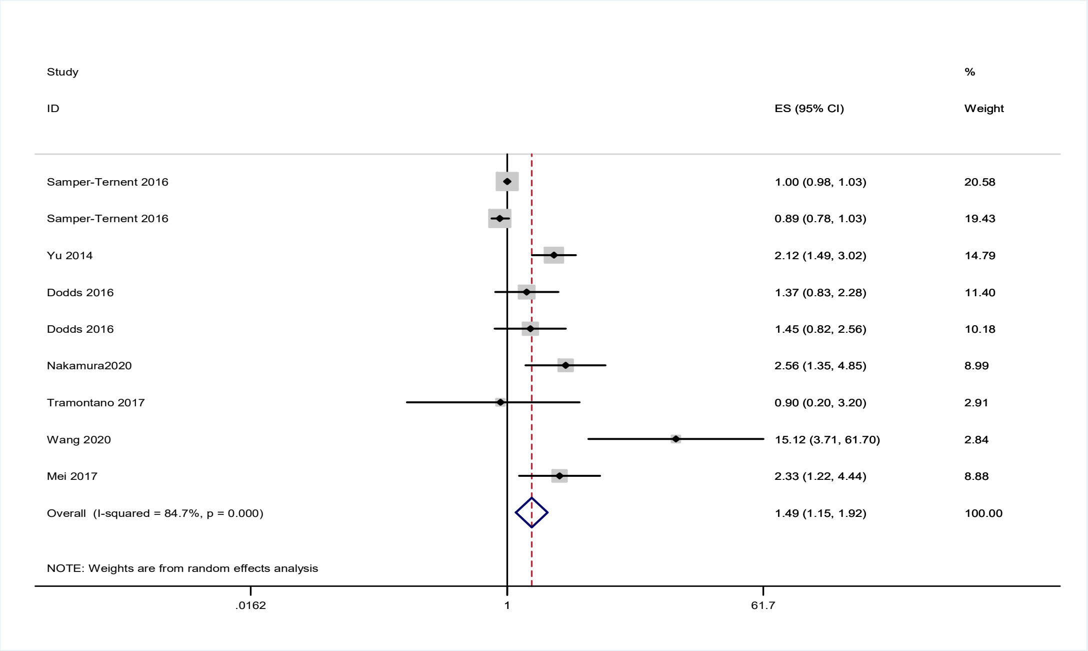

Figure S5. Forest plot of the association between female and sarcopenia.

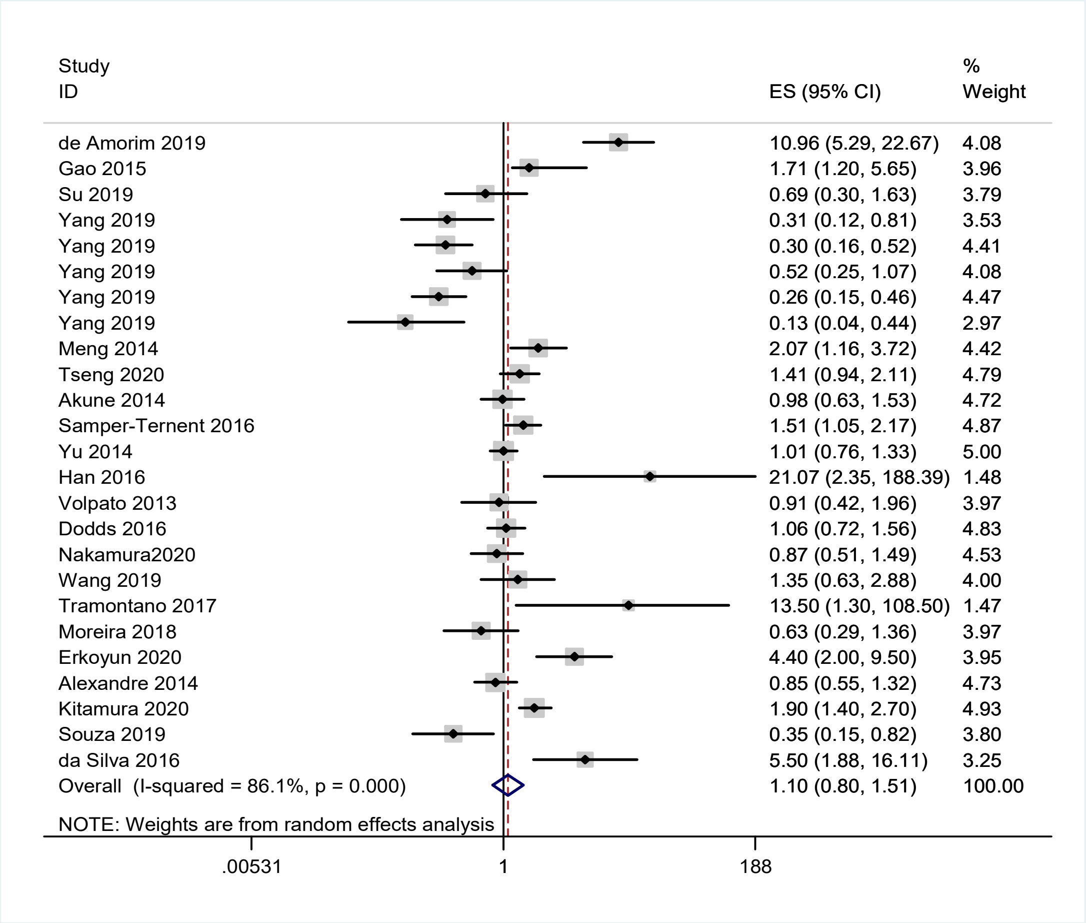

Figure S6. Forest plot of the association between male and sarcopenia.

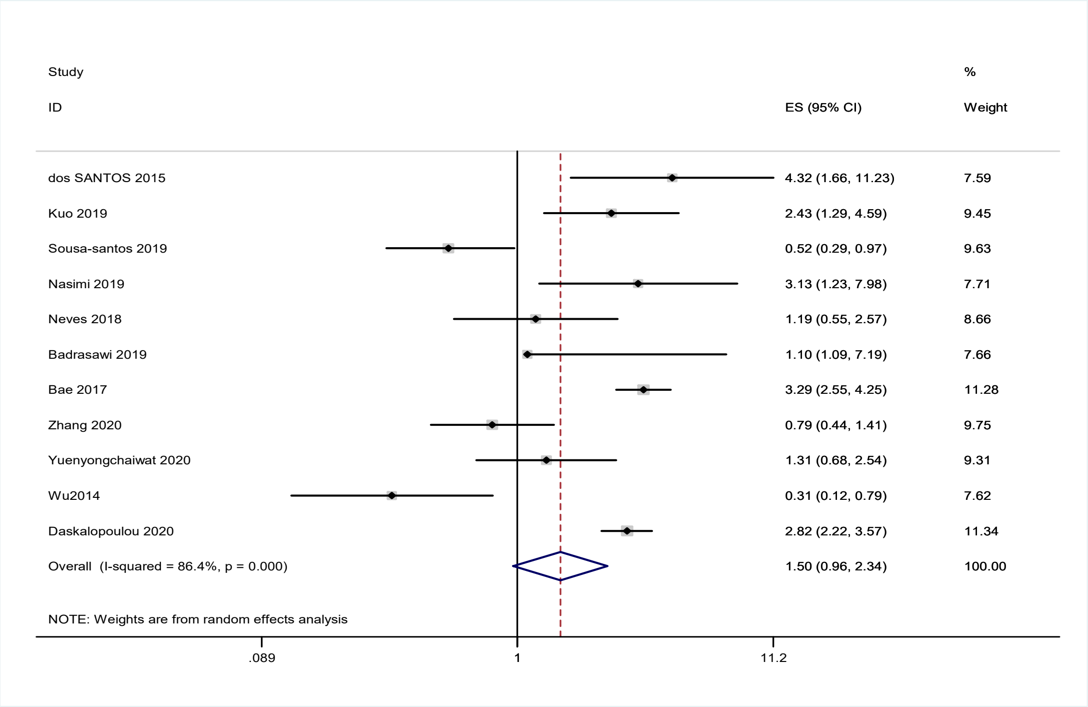

Figure S7. Forest plot of the association between higher level of education and sarcopenia.

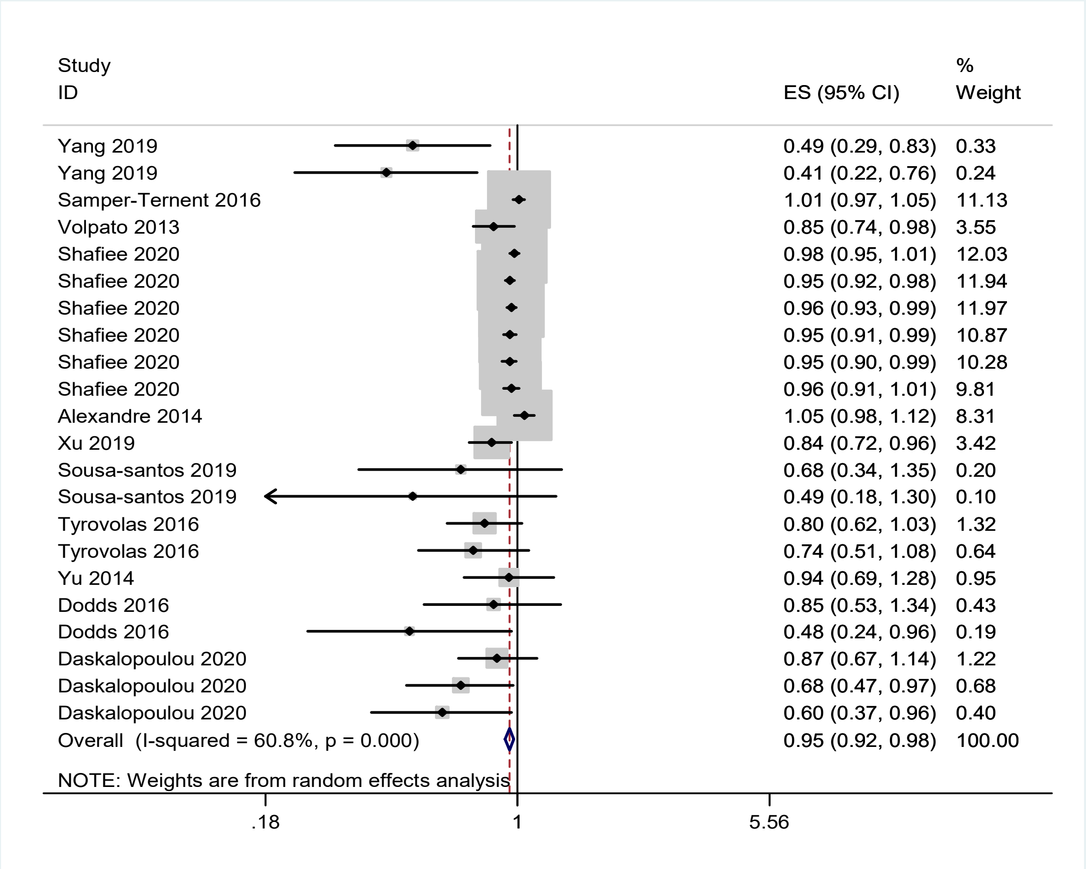

Figure S8. Forest plot of the association between higher BMI (overweight/obesity) and sarcopenia.

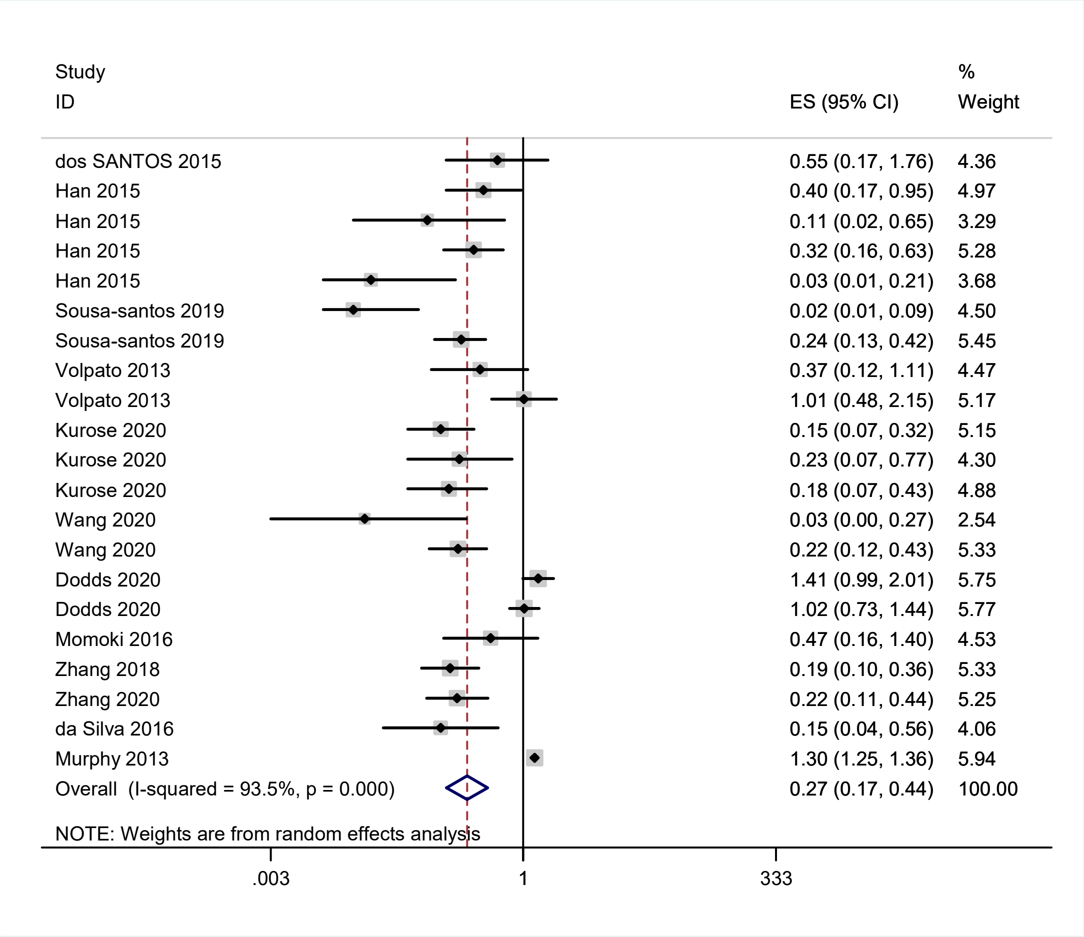

Figure S9. Forest plot of the association between smoking and sarcopenia.

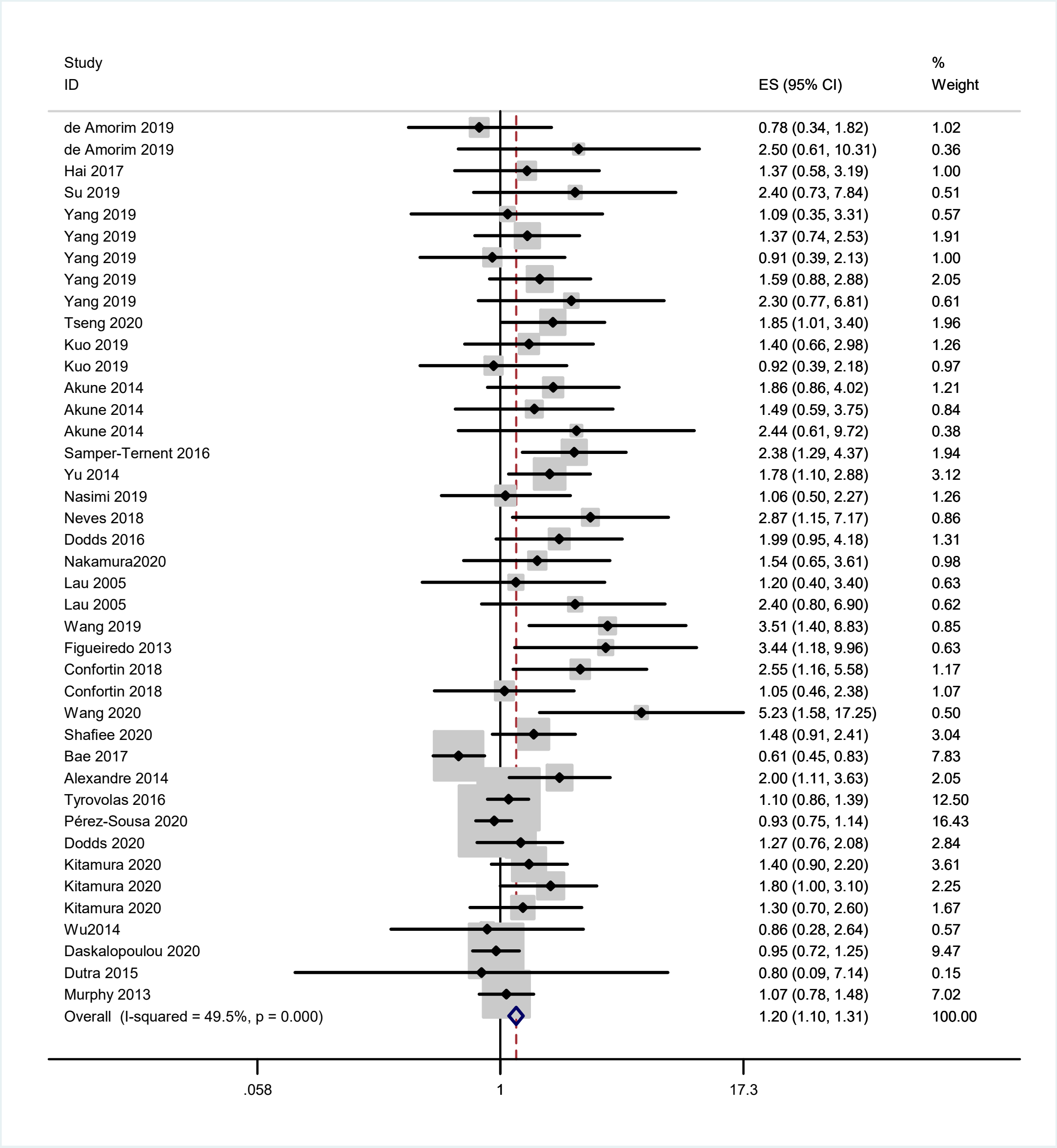

Figure S10. Forest plot of the association between malnutrition/malnutrition risk and sarcopenia.

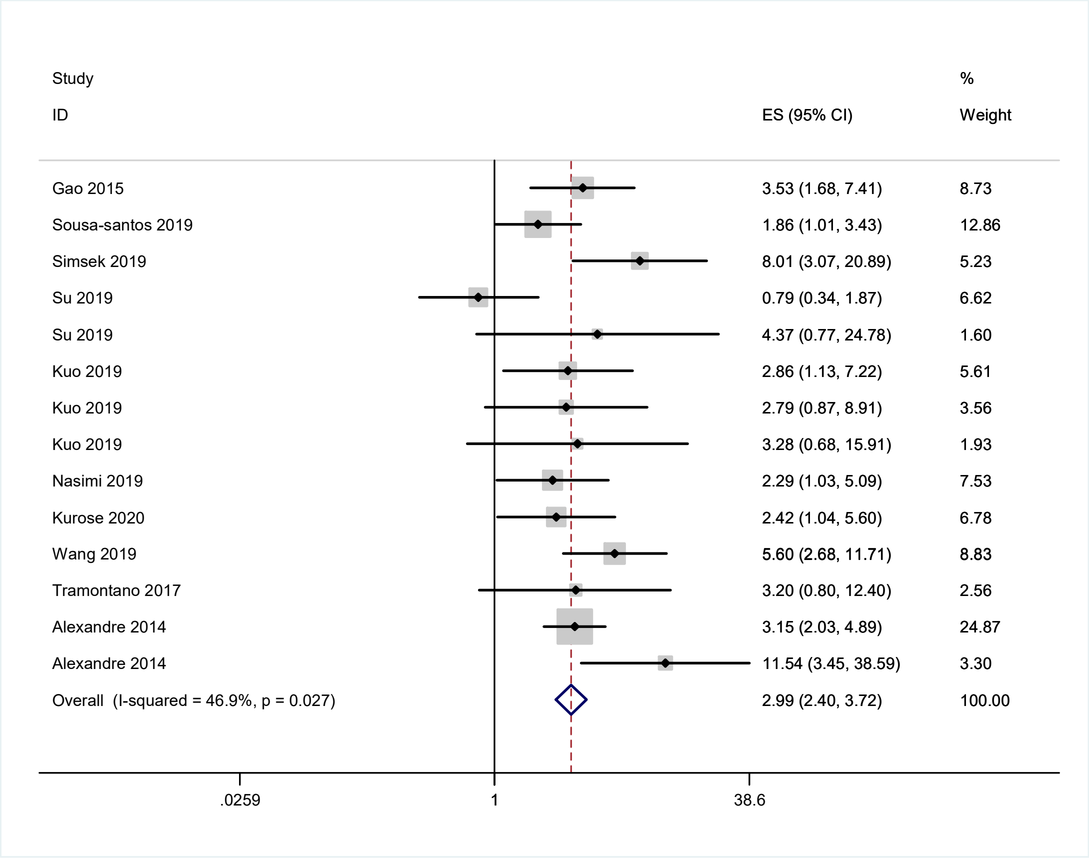

Figure S11. Forest plot of the association between short sleeping time and sarcopenia.

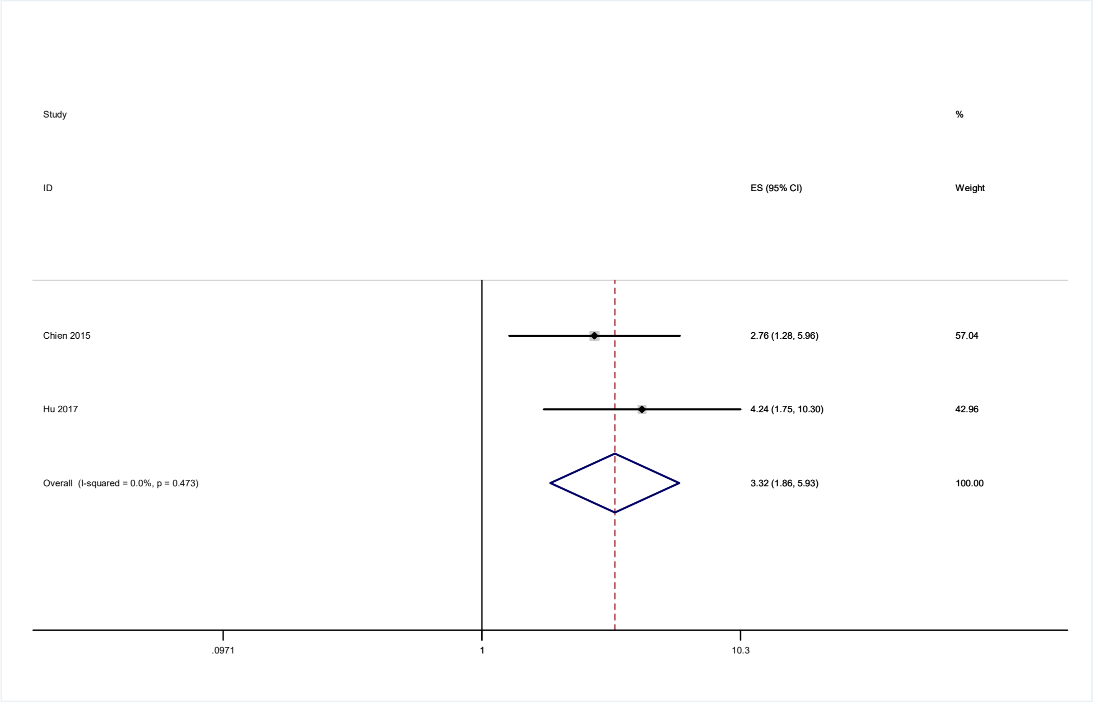

Figure S12. Forest plot of the association between long sleeping time and sarcopenia.

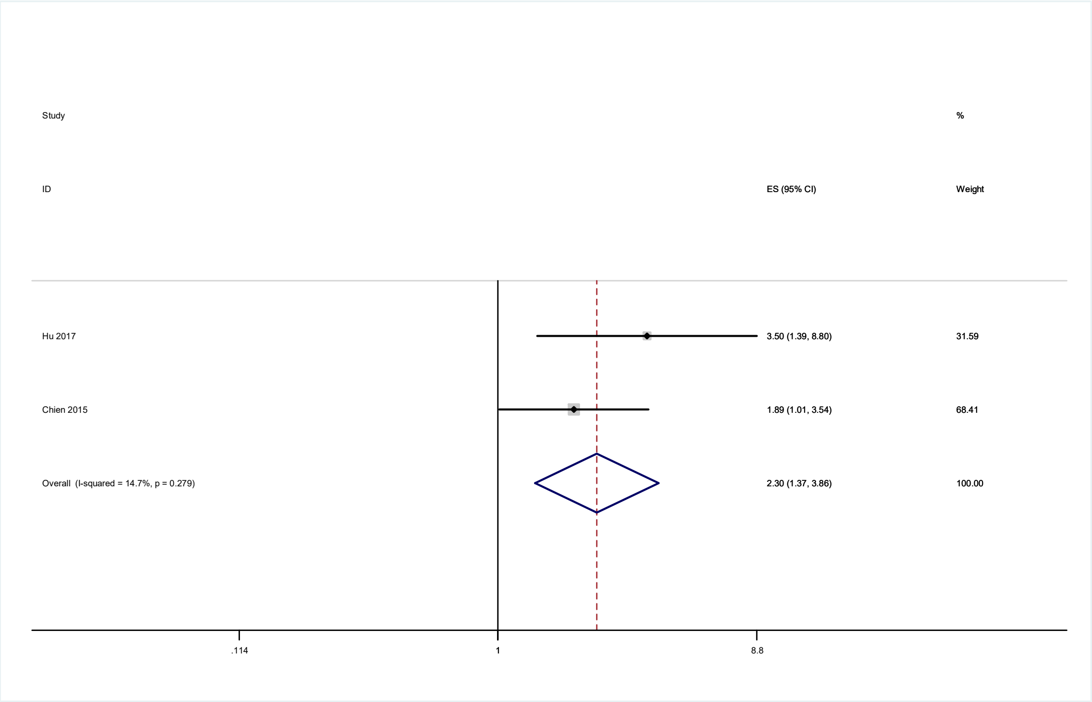

Figure S13. Forest plot of the association between living alone and sarcopenia.

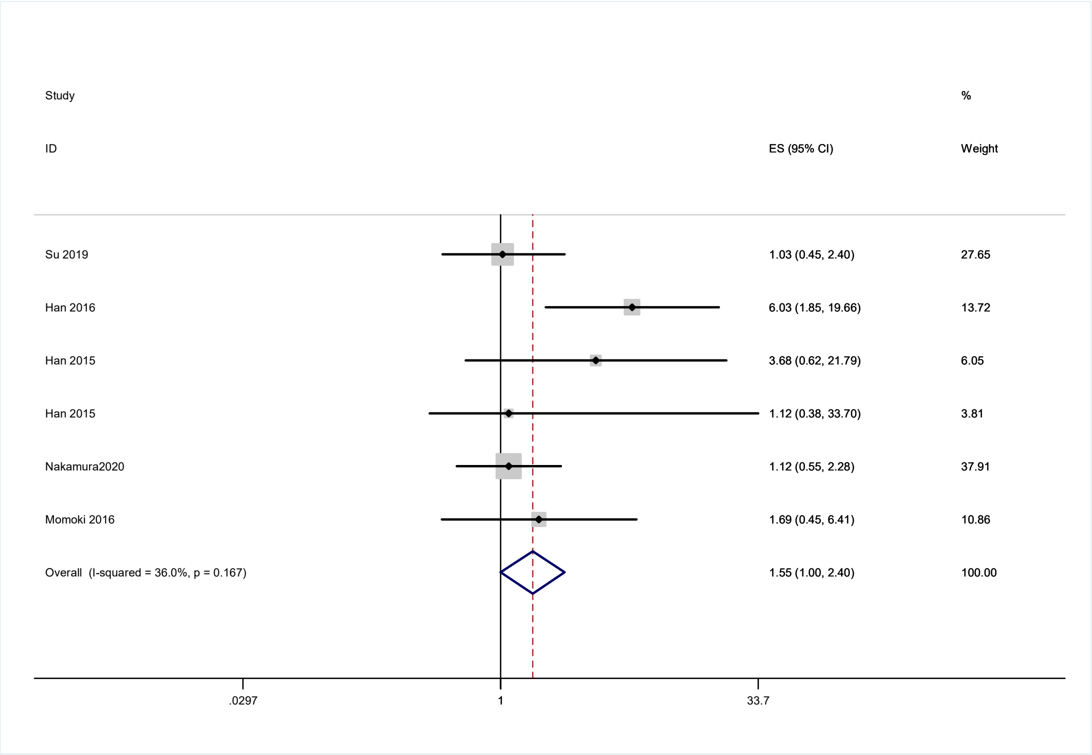

Figure S14. Forest plot of the association between physical inactivity and sarcopenia.

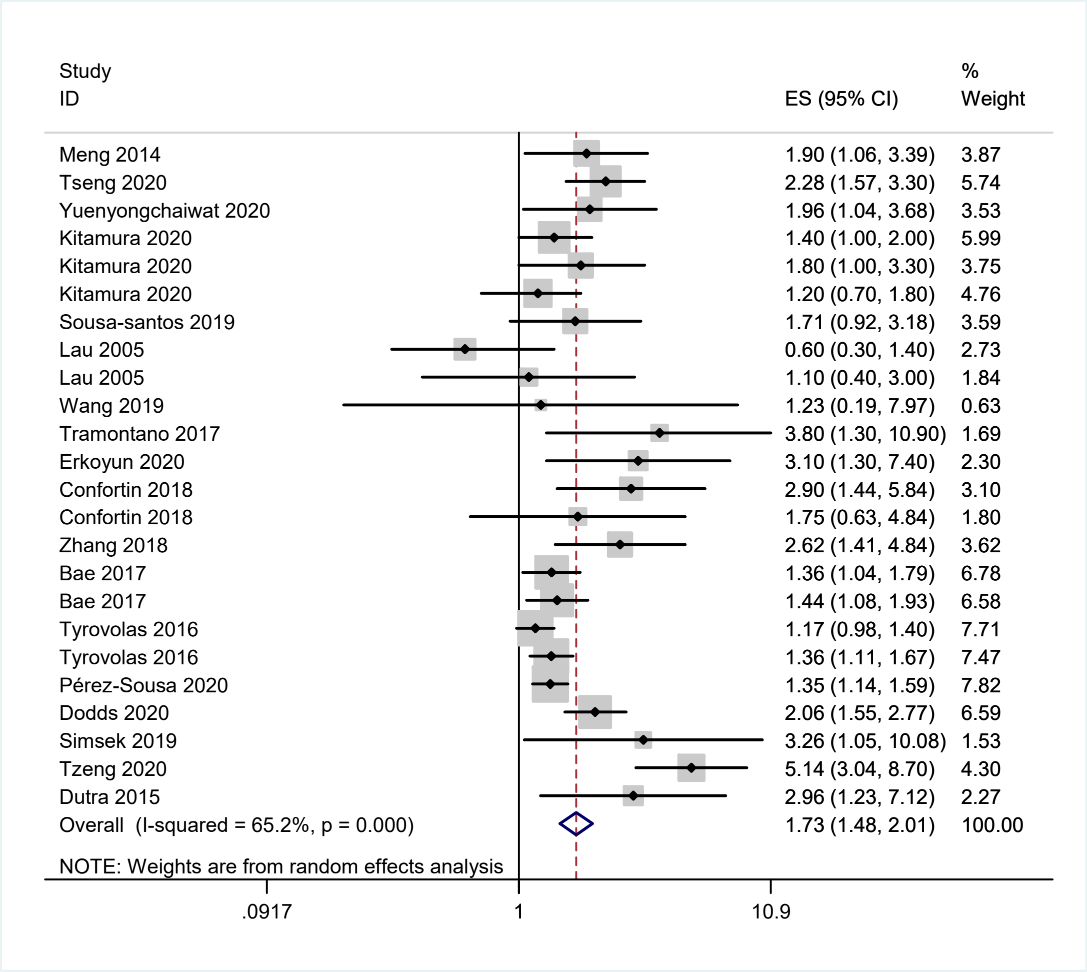

Figure S15. Forest plot of the association between drinking and sarcopenia.

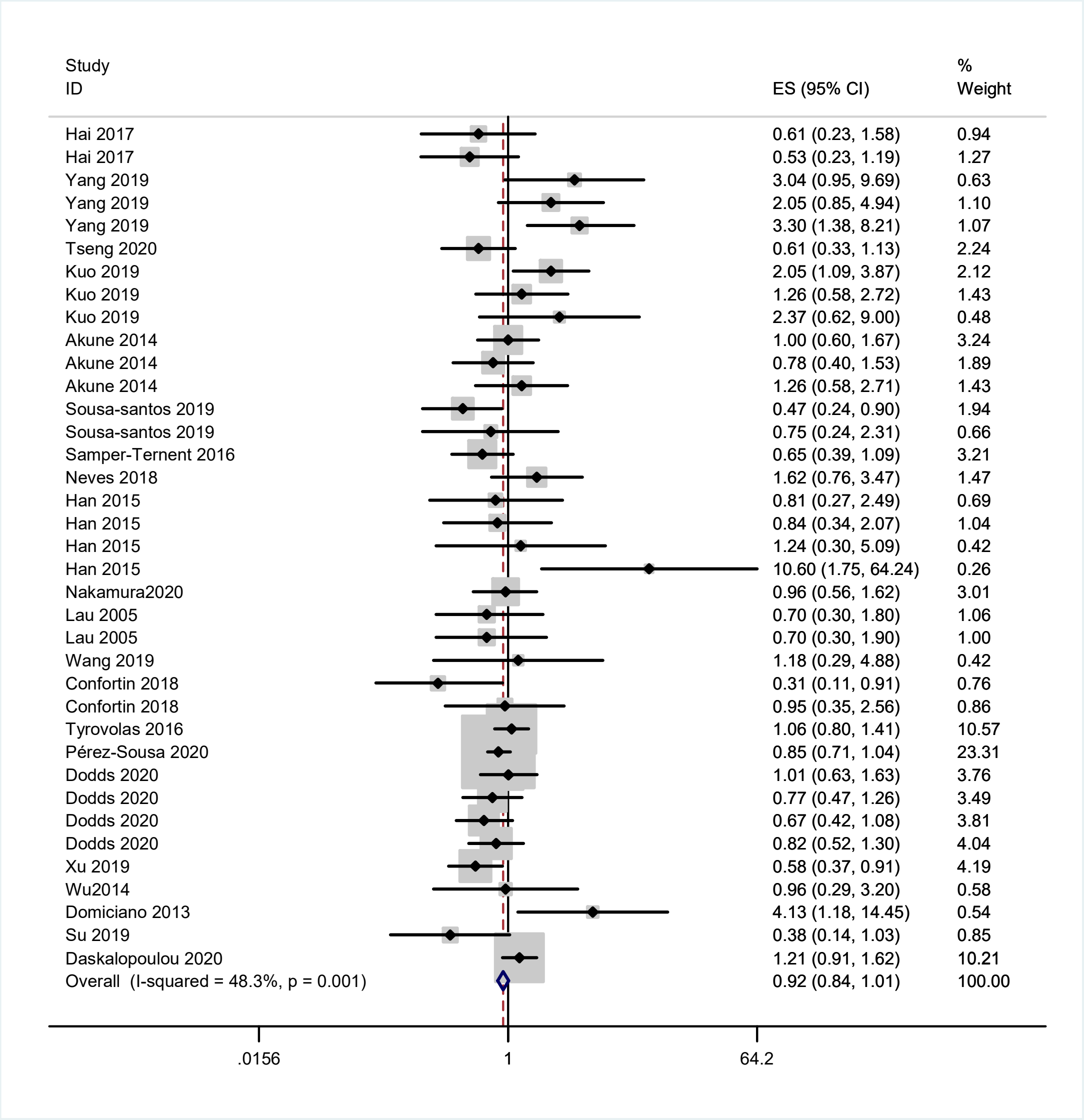

Figure S16. Forest plot of the association between diabetes and sarcopenia.

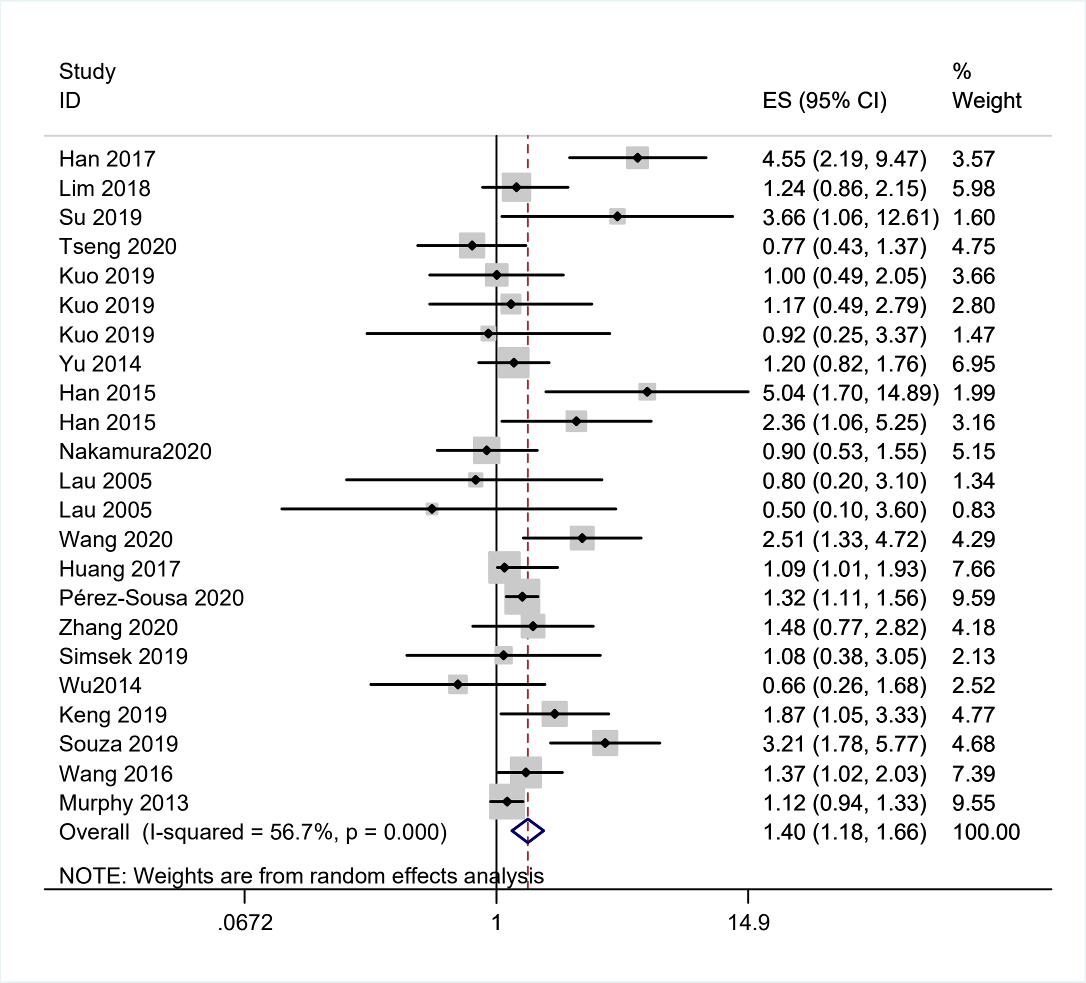

Figure S17. Forest plot of the association between cognitive impairment and sarcopenia.

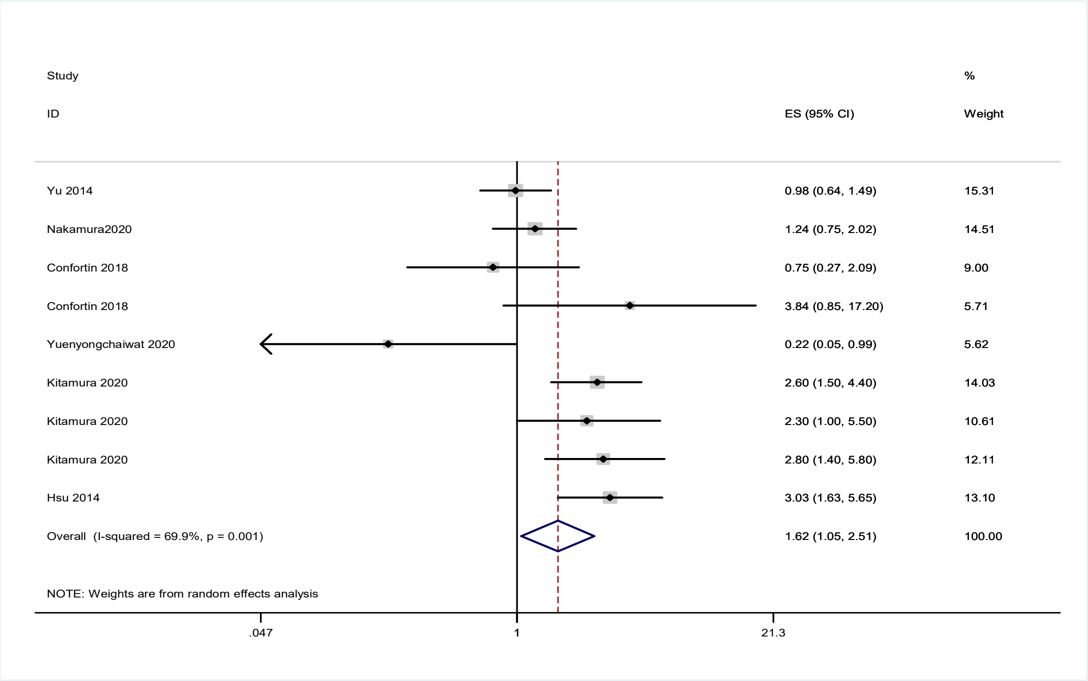

Figure S18. Forest plot of the association between heart diseases and sarcopenia.

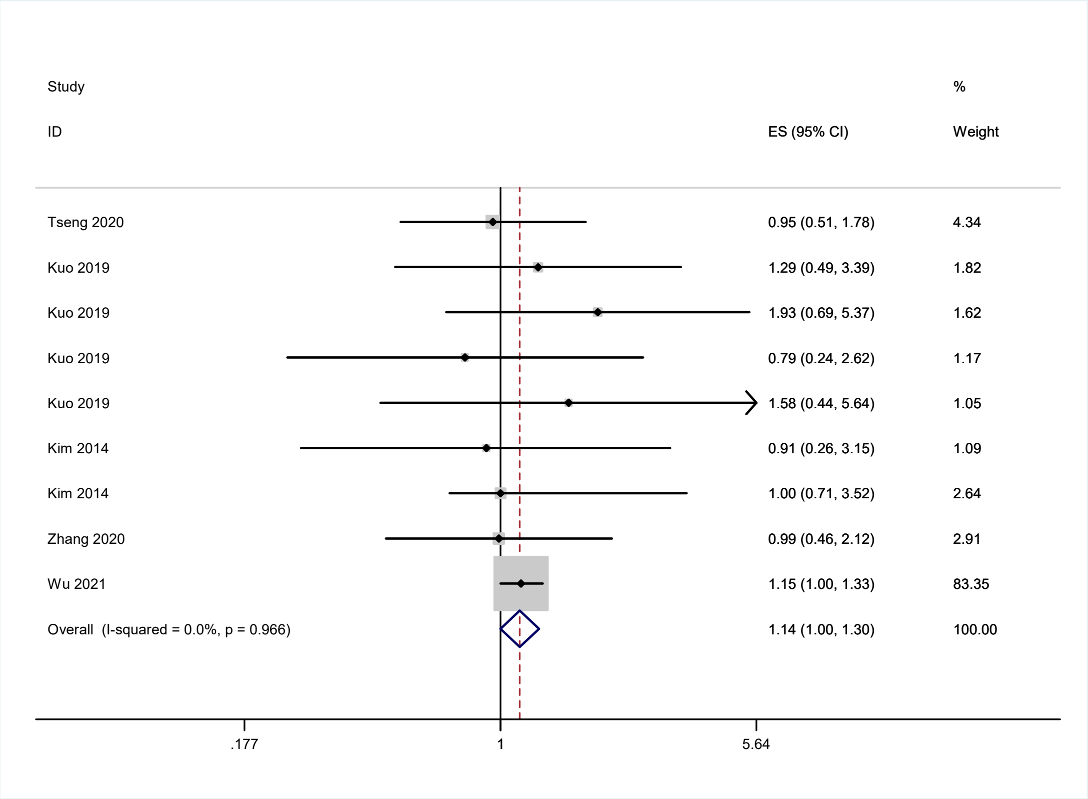

Figure S19. Forest plot of the association between respiratory diseases and sarcopenia.

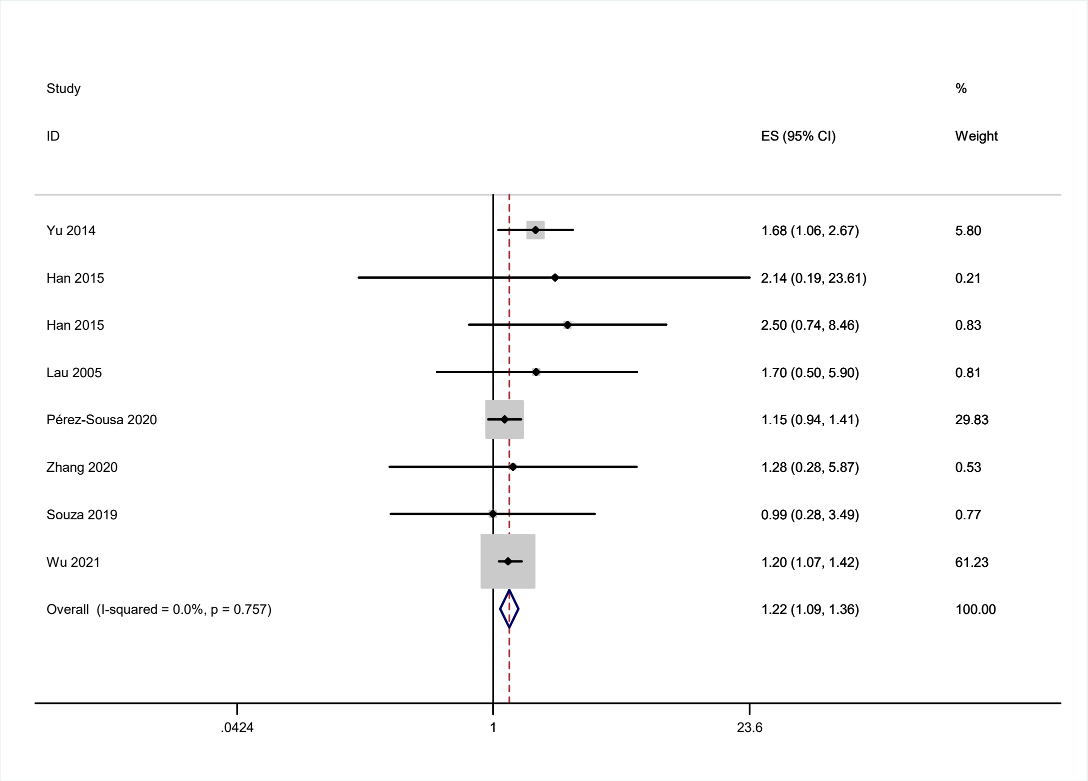

Figure S20. Forest plot of the association between osteopenia/osteoporosis and sarcopenia.

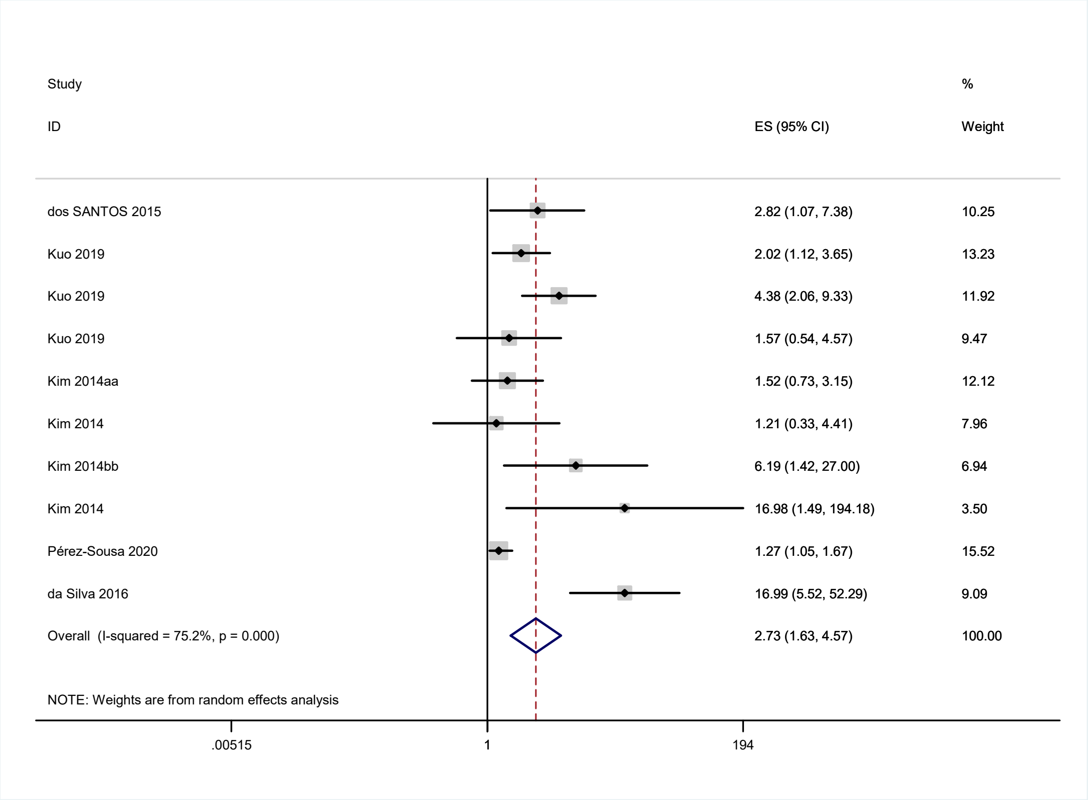

Figure S21. Forest plot of the association between osteoarthritis and sarcopenia.

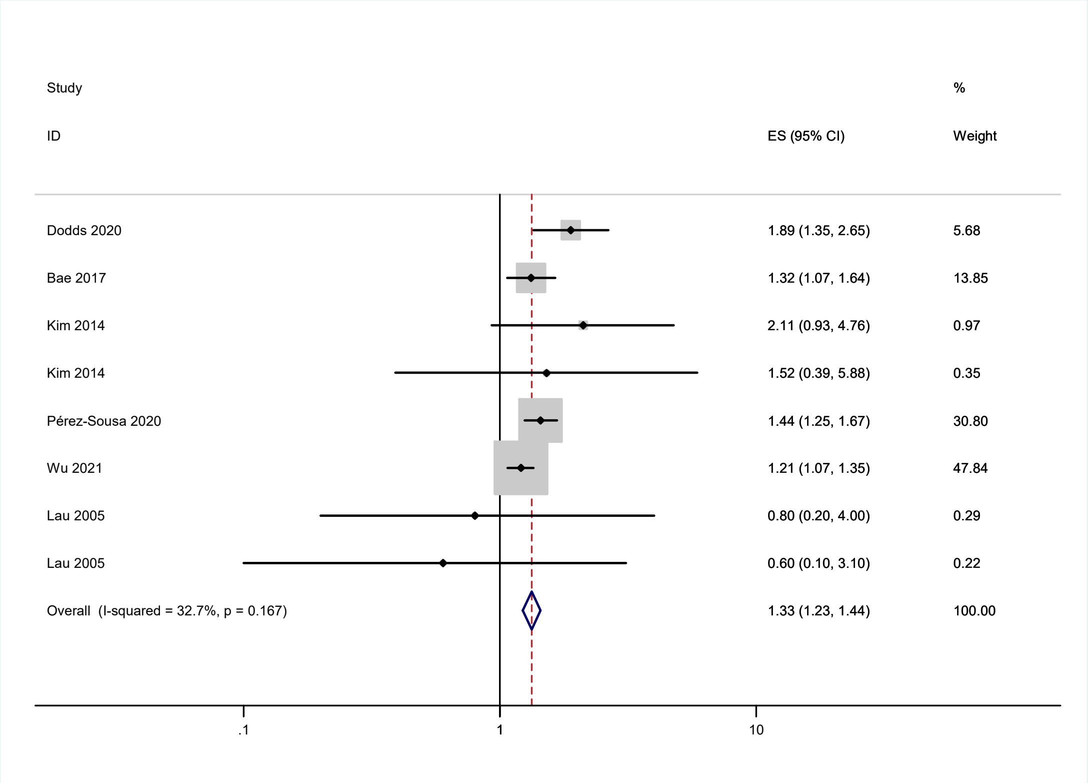

Figure S22. Forest plot of the association between depression and sarcopenia.

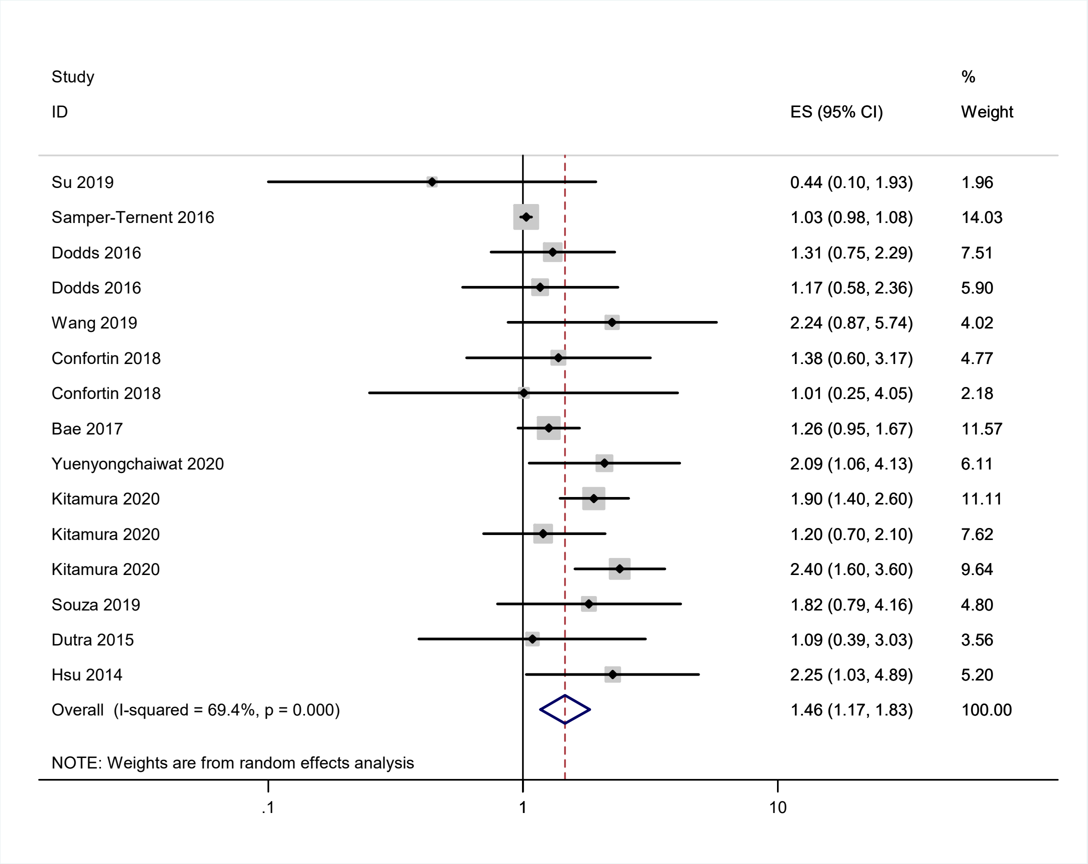

Figure S23. Forest plot of the association between fall and sarcopenia.

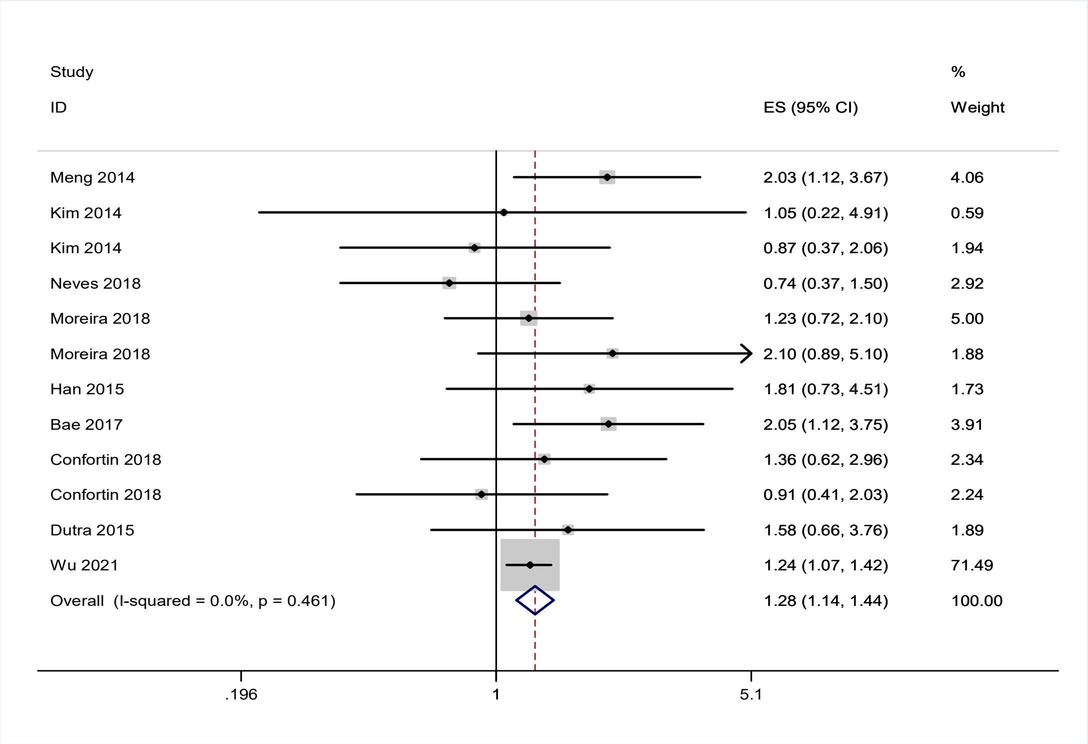

Figure S24. Forest plot of the association between anorexia and sarcopenia.

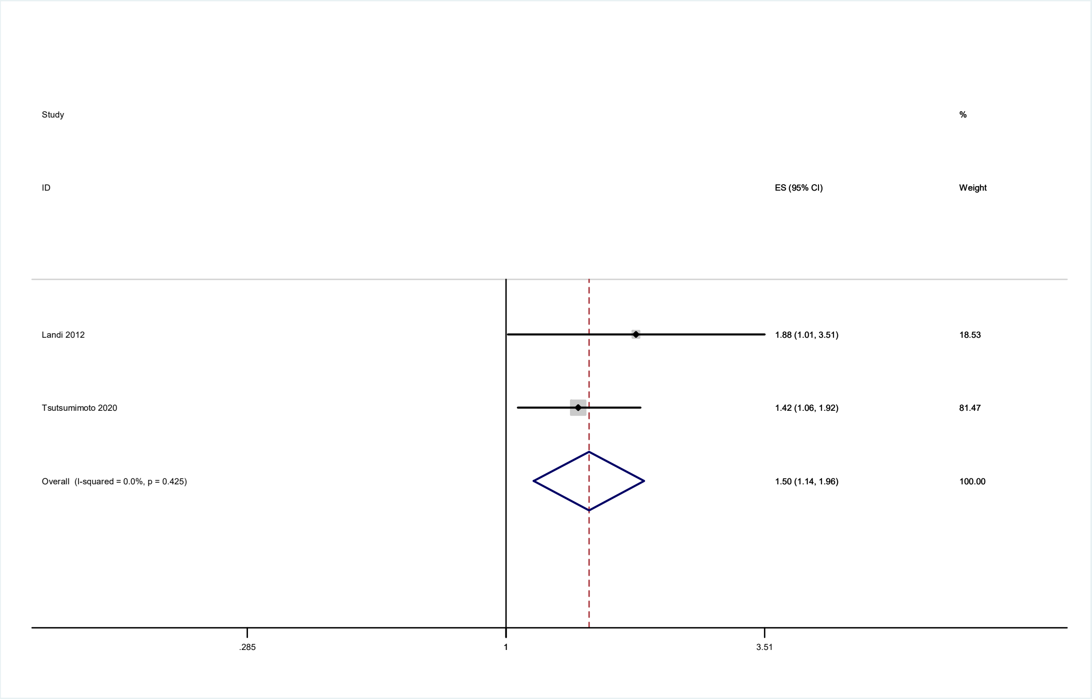

Figure S25. Forest plot of the association between anemia and sarcopenia.

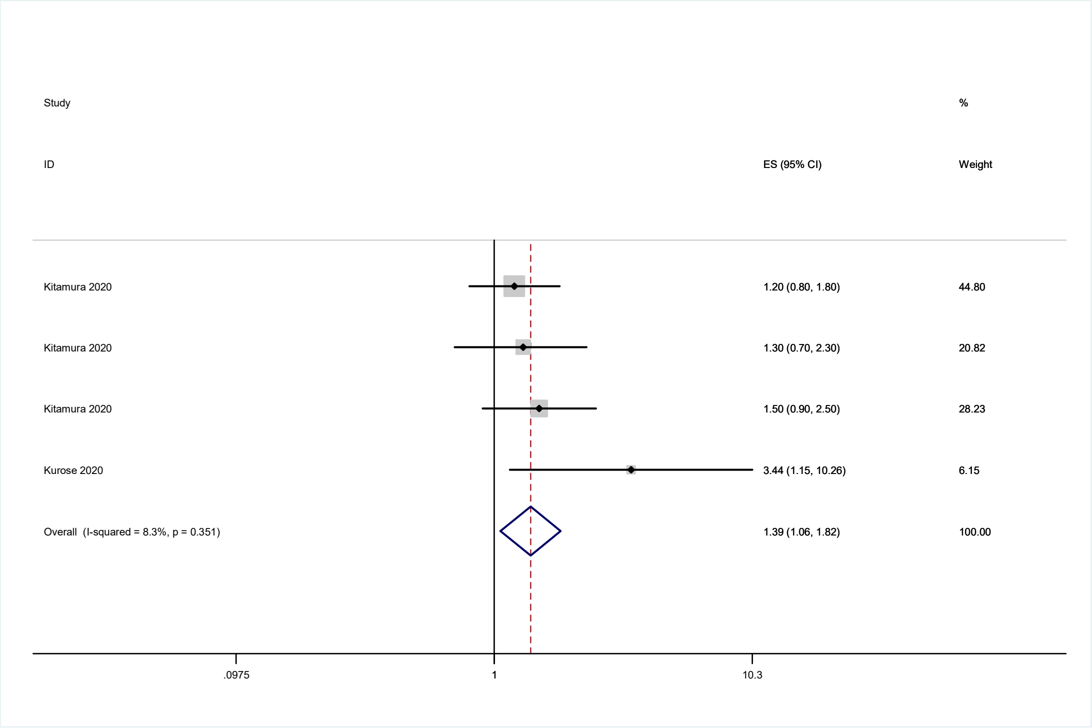

Figure S26. Forest plot of the association between hypertension and sarcopenia.

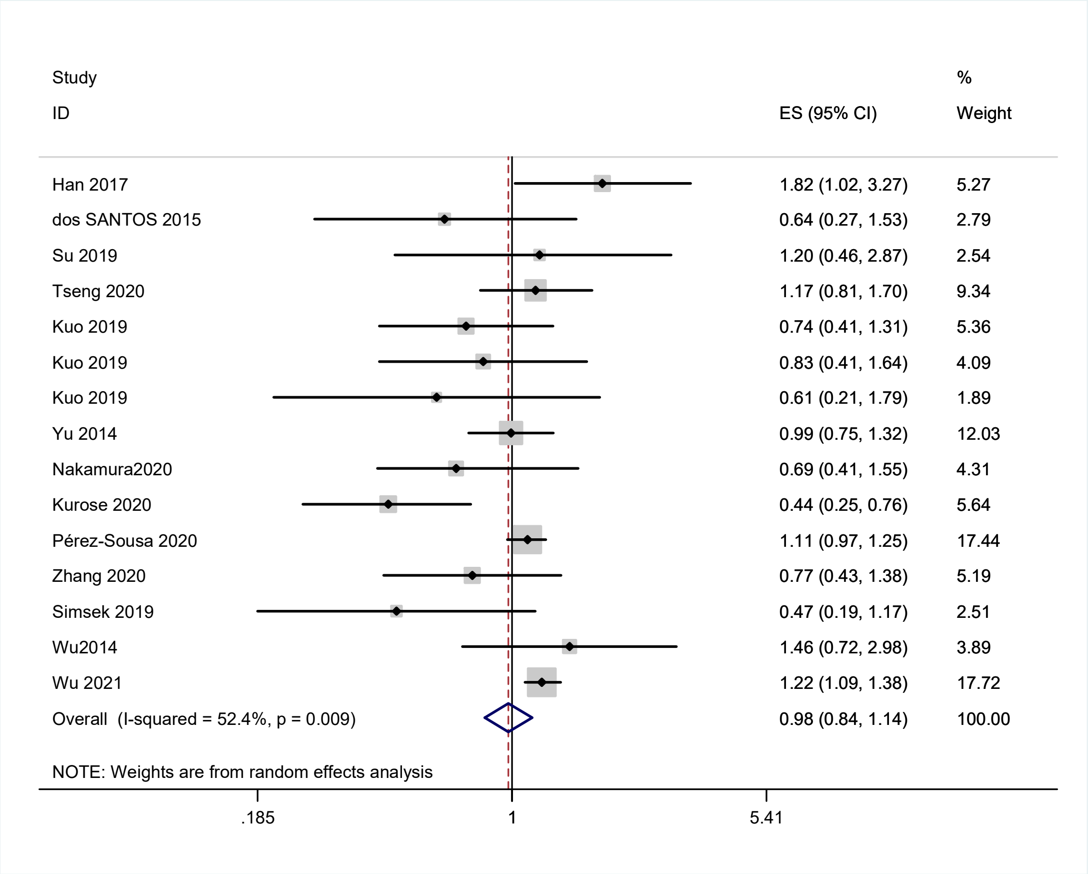

Figure S27. Forest plot of the association between hyperlipidemia and sarcopenia.

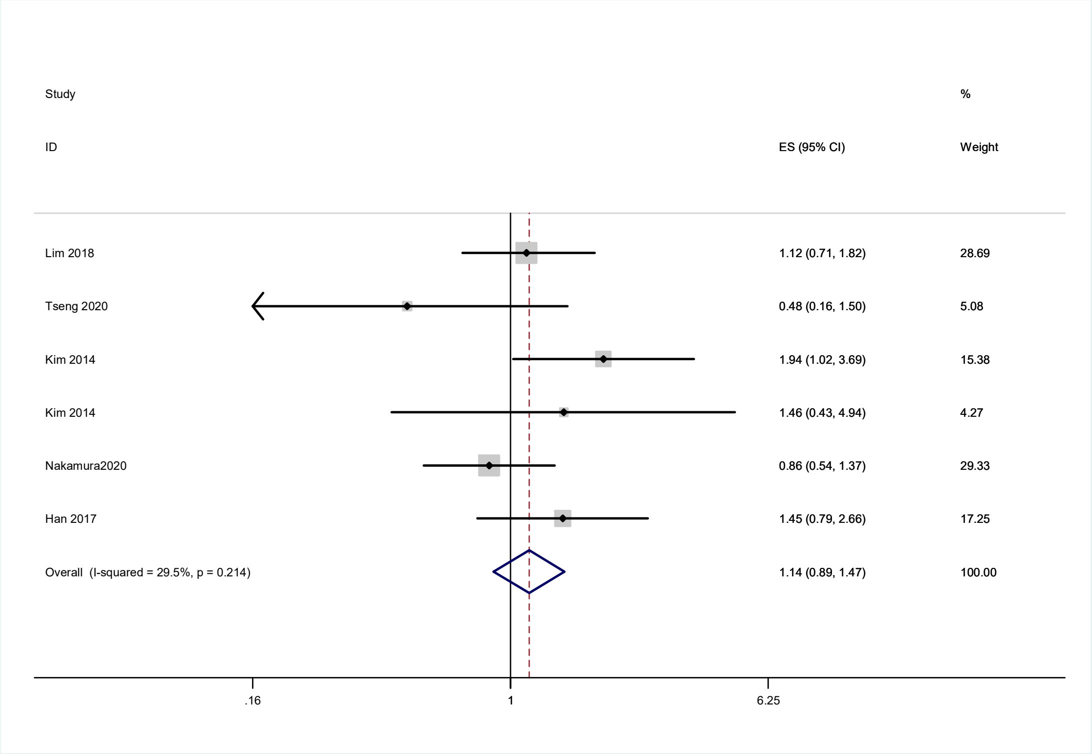

Figure S28. Forest plot of the association between stroke and sarcopenia.

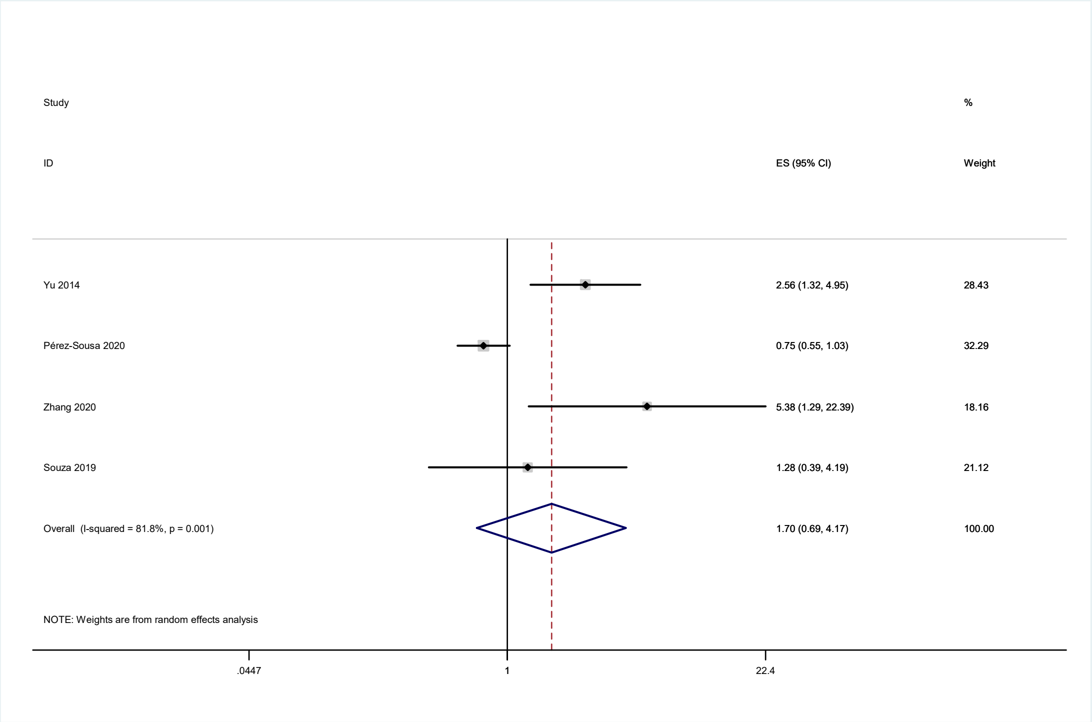

Figure S29. Forest plot of the association between cancer and sarcopenia.

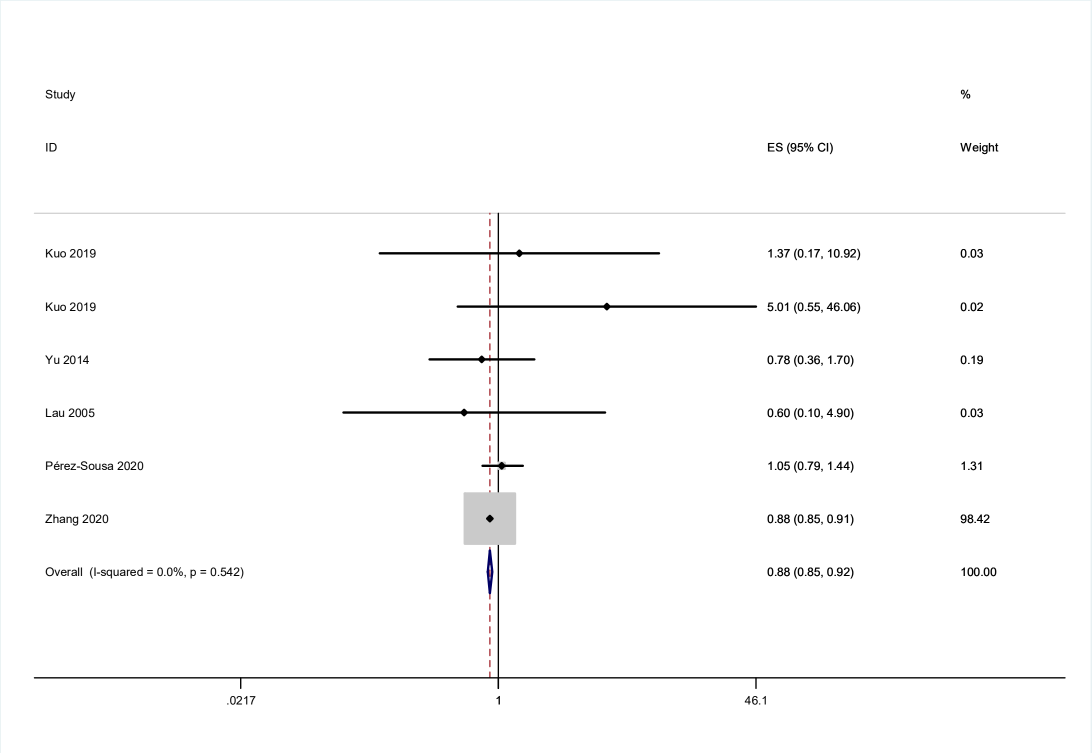

Figure S30. Forest plot of the association between pain and sarcopenia.

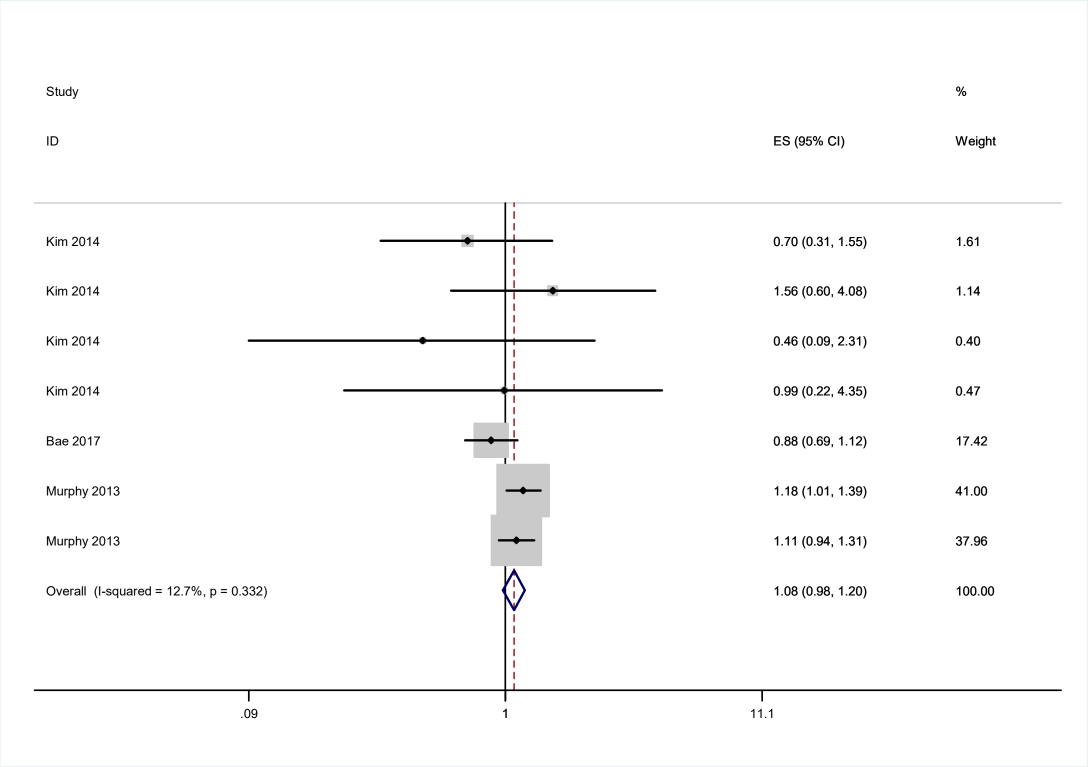

Figure S31. Forest plot of the association between liver disease and sarcopenia.

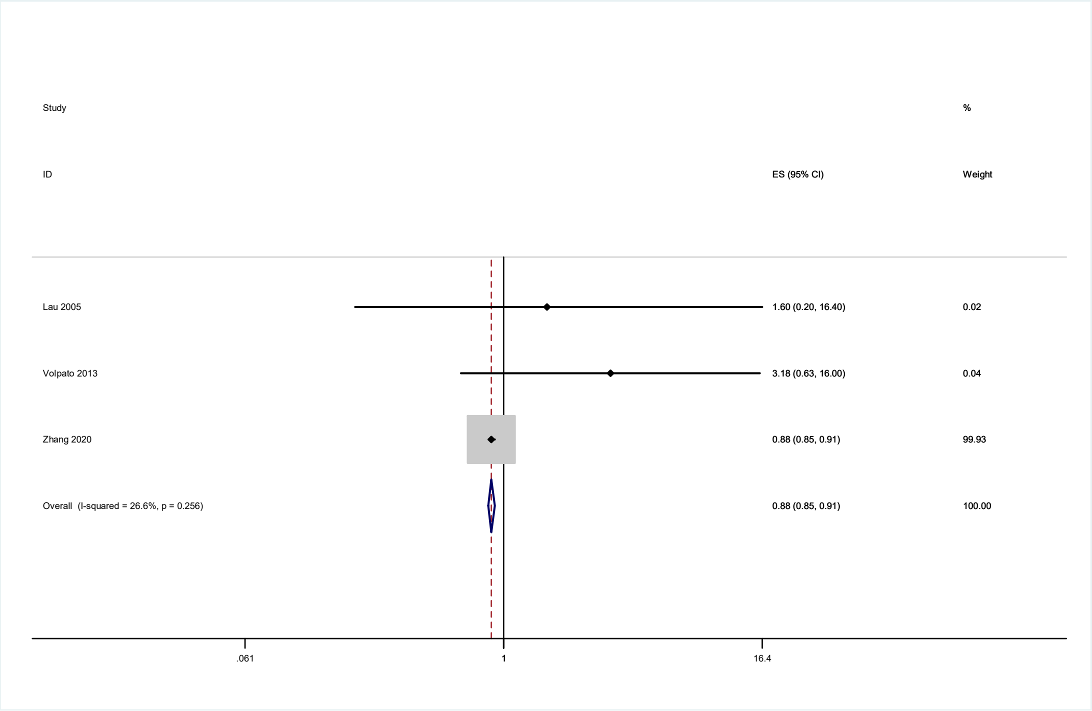

Figure S32. Forest plot of the association between kidney disease and sarcopenia.

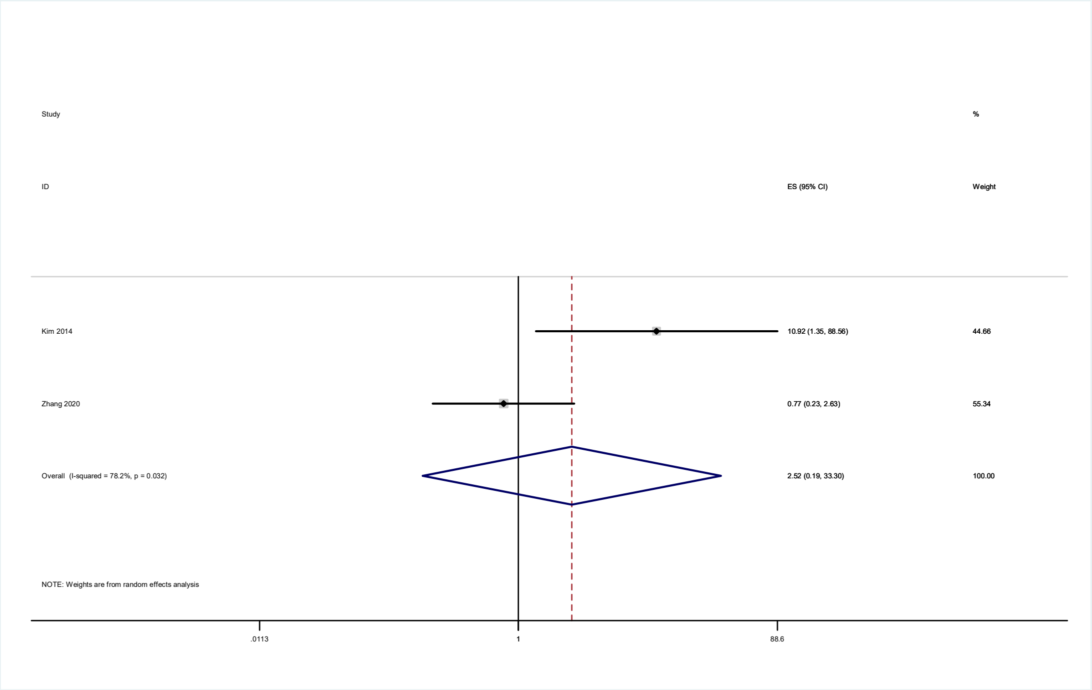

Figure S33. Funnel plot of the association between male and sarcopenia.

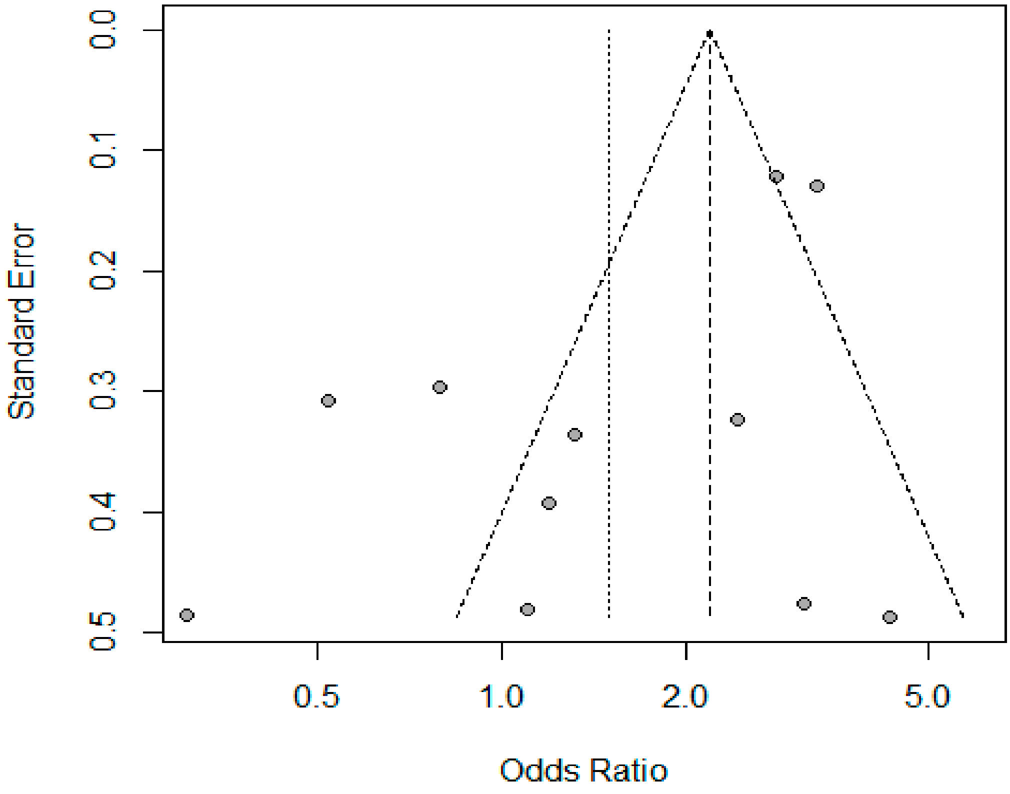

Figure S34. Funnel plot of the association between overweight/obesity and sarcopenia.

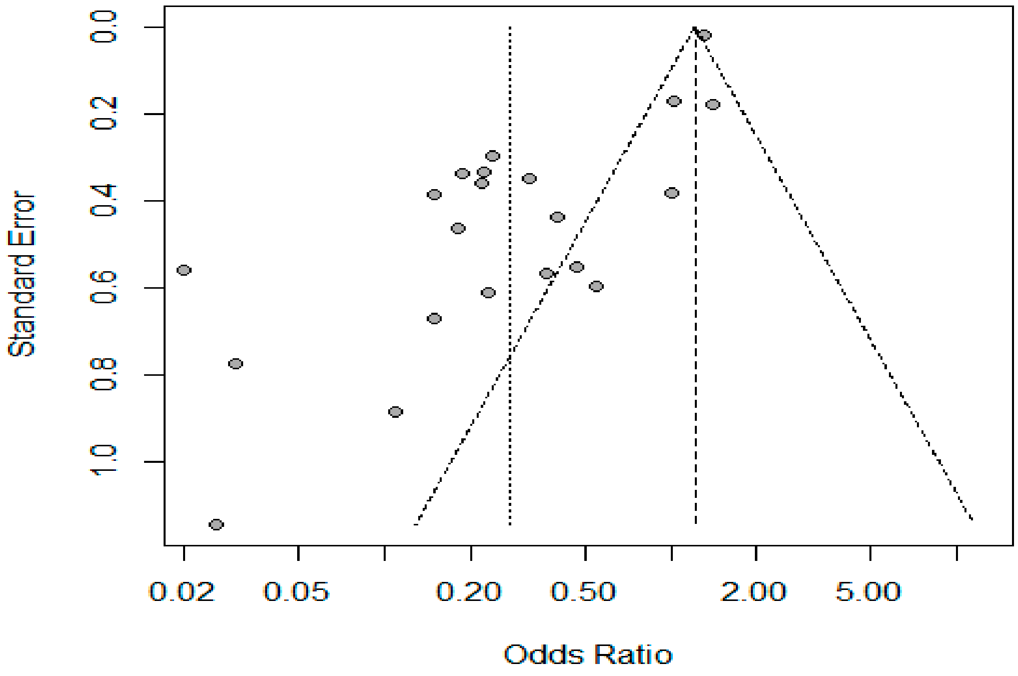

Figure S35. Funnel plot of the association between underweight and sarcopenia.

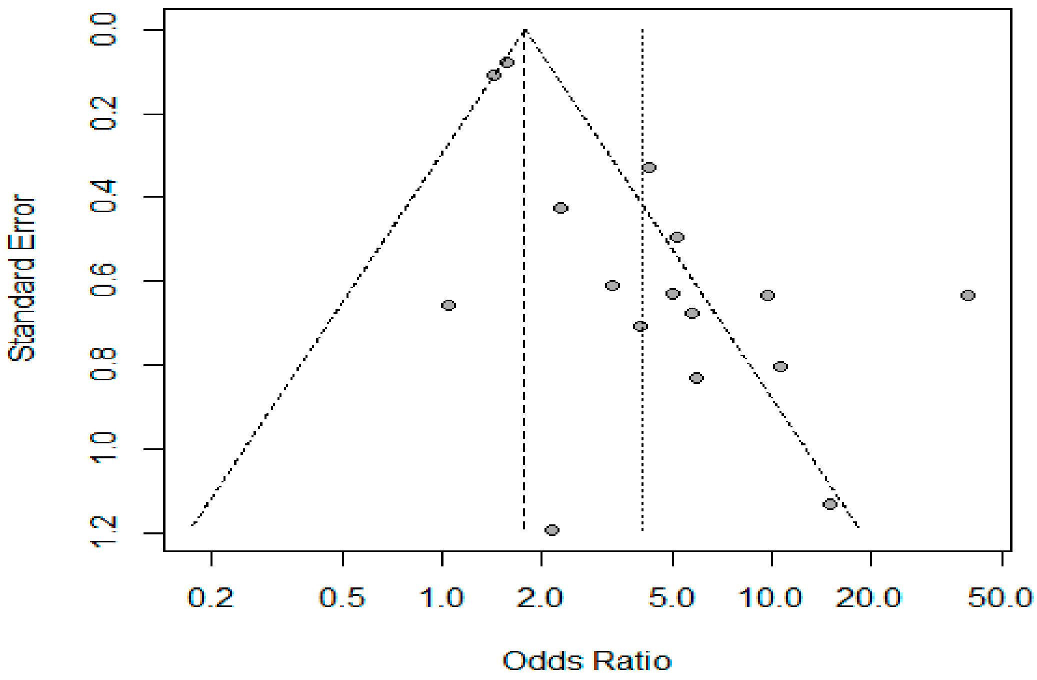

Figure S36. Funnel plot of the association between high level of education and sarcopenia.

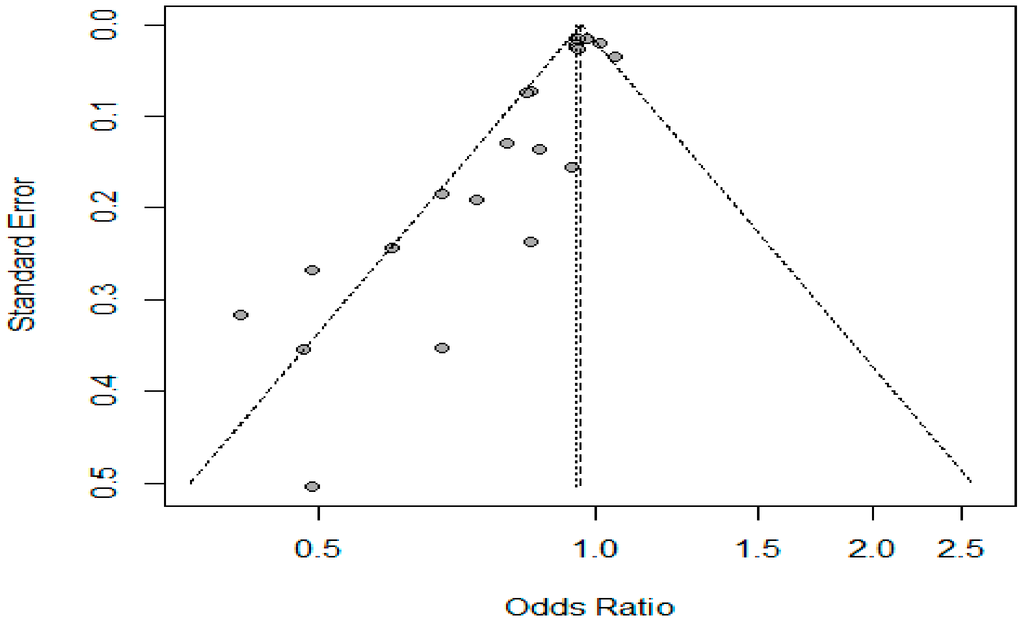

Figure S37. Funnel plot of the association between smoking and sarcopenia.

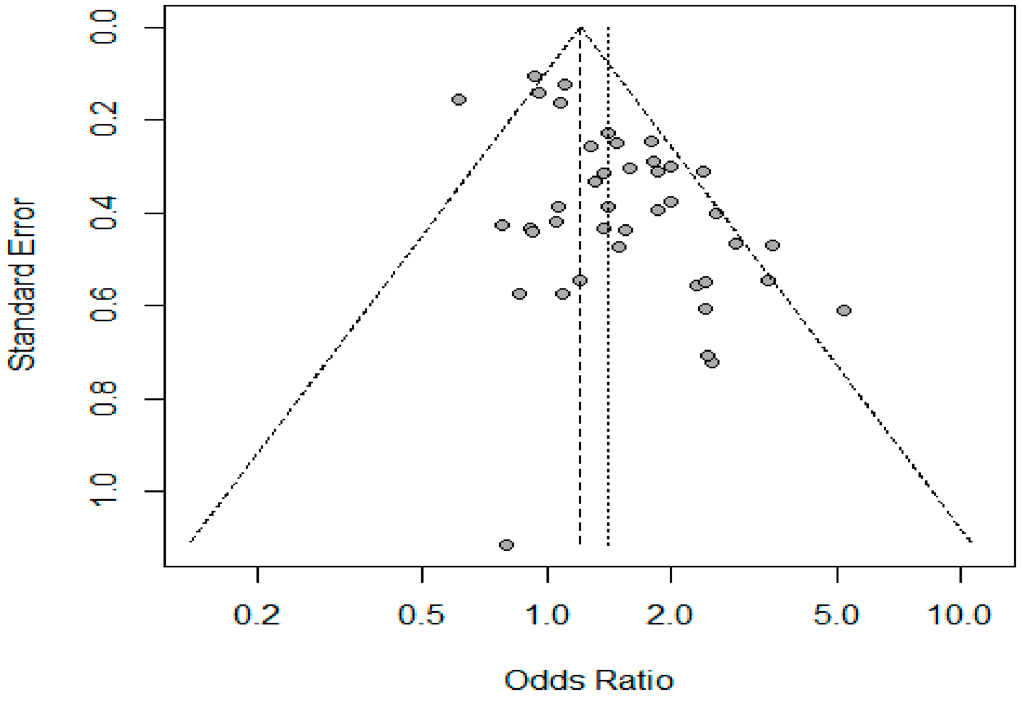

Figure S38. Funnel plot of the association between physical inactivity and sarcopenia.

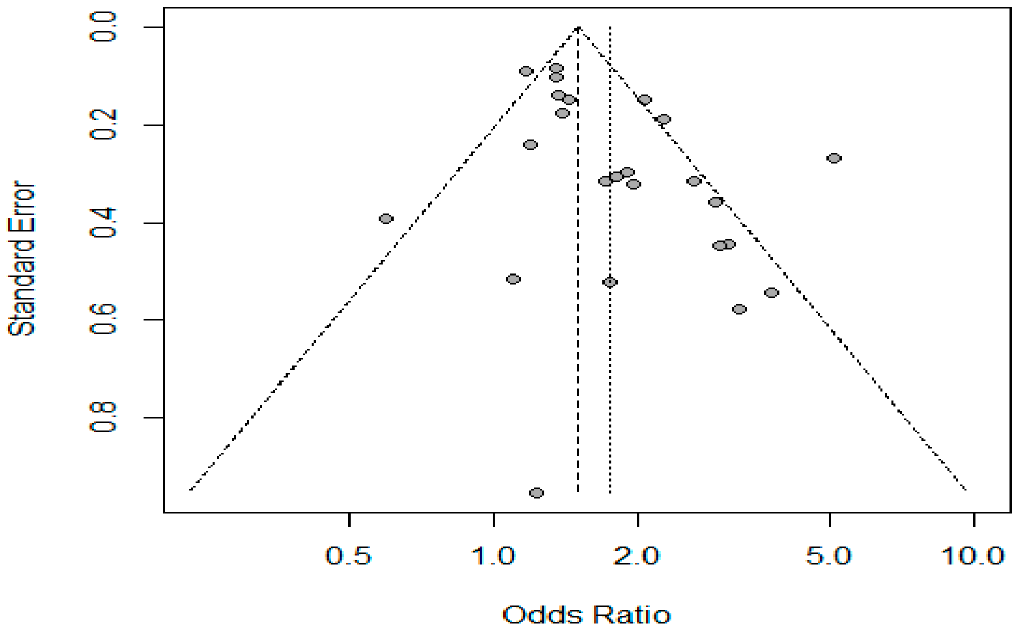

Figure S39. Funnel plot of the association between hypertension and sarcopenia.

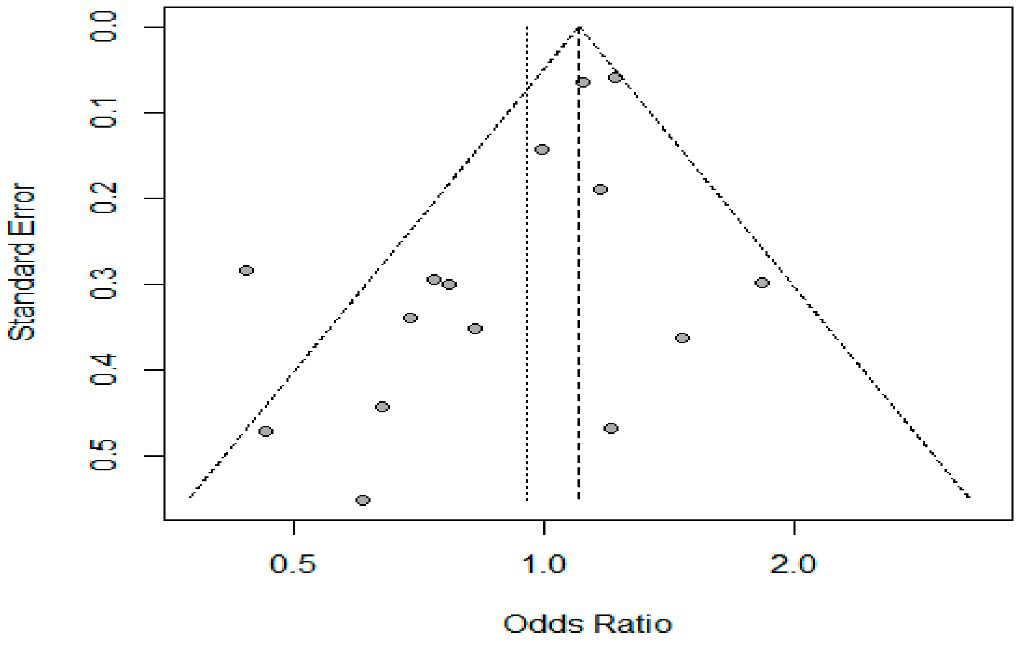

Figure S40. Funnel plot of the association between depression and sarcopenia.

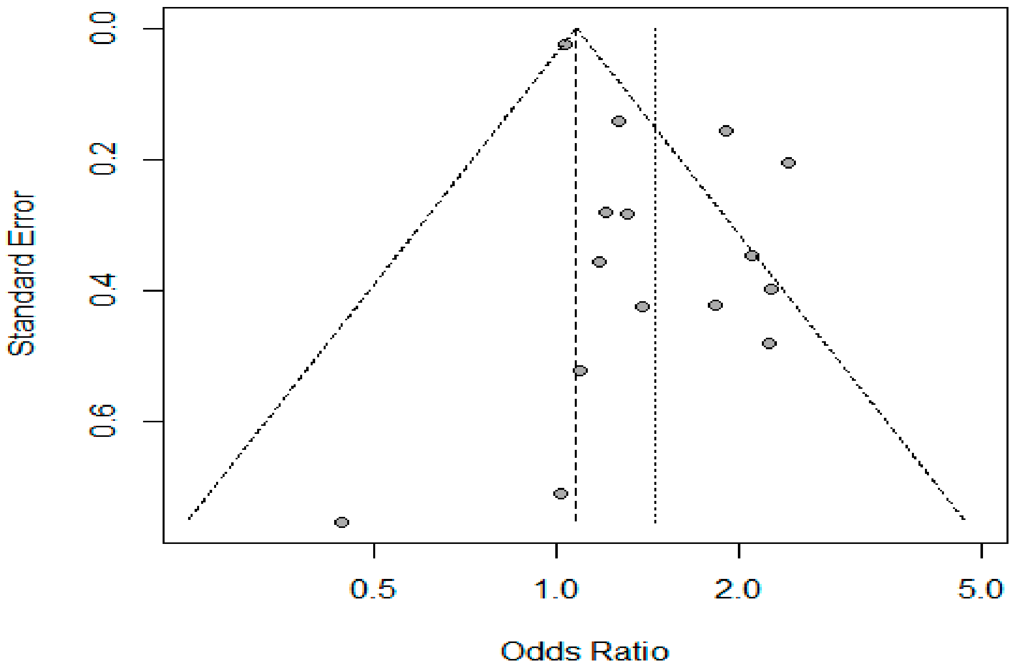

Figure S41. Funnel plot of the association between age and sarcopenia.

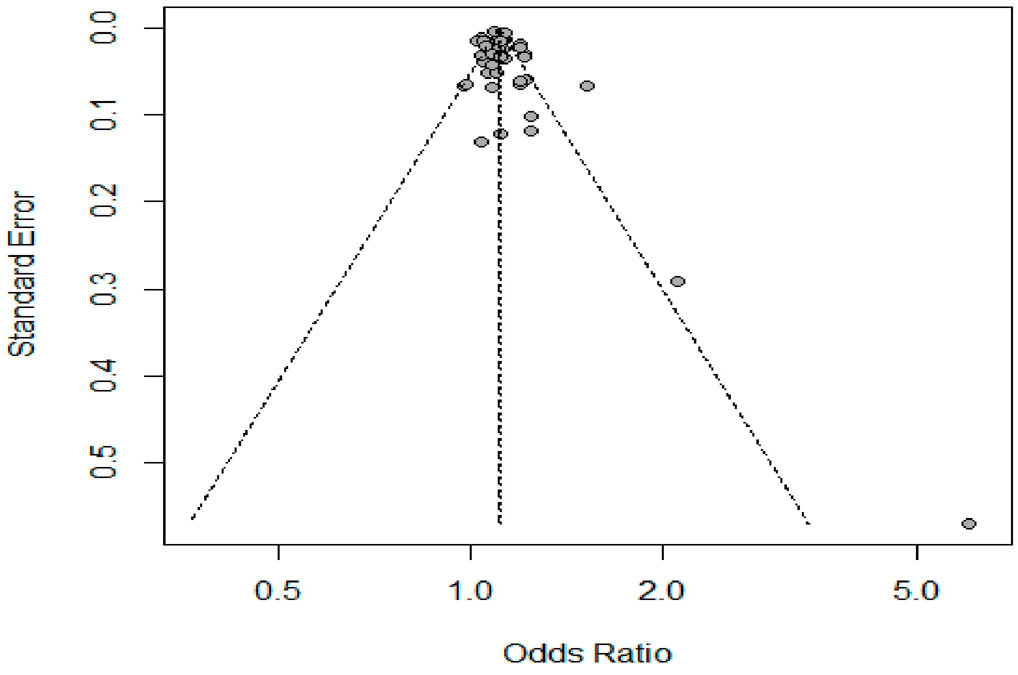

Figure S42. Funnel plot of the association between female and sarcopenia.

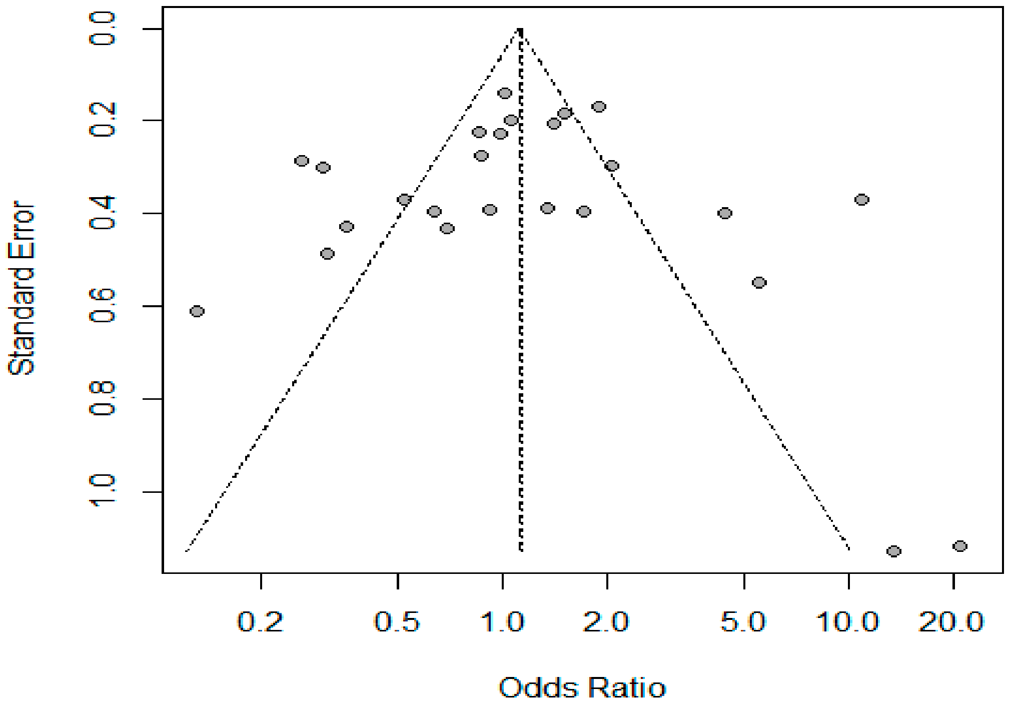

Figure S43. Funnel plot of the association between malnutrition/malnutrition risk and sarcopenia.

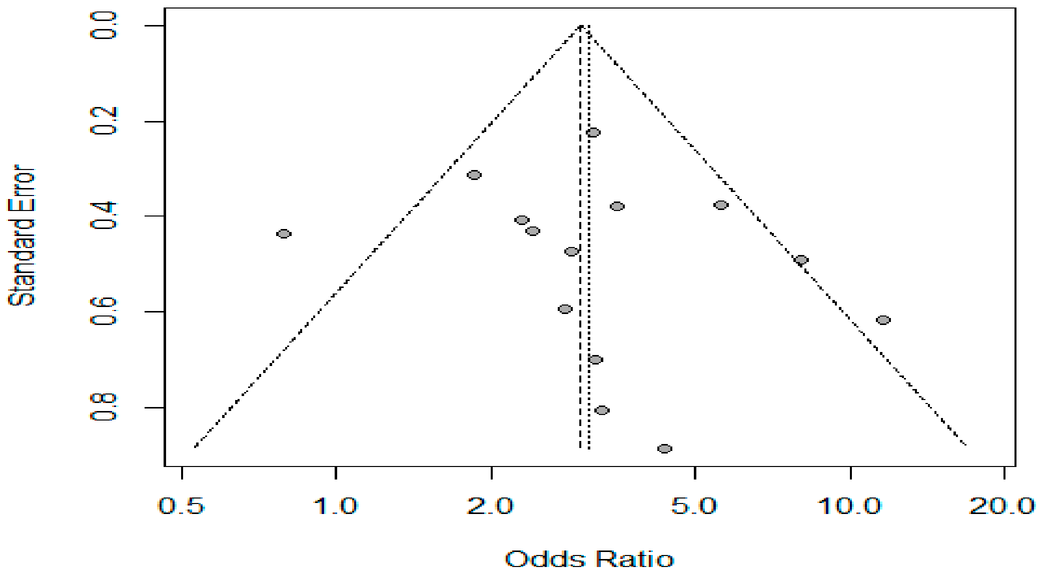

Figure S44. Funnel plot of the association between drinking and sarcopenia.

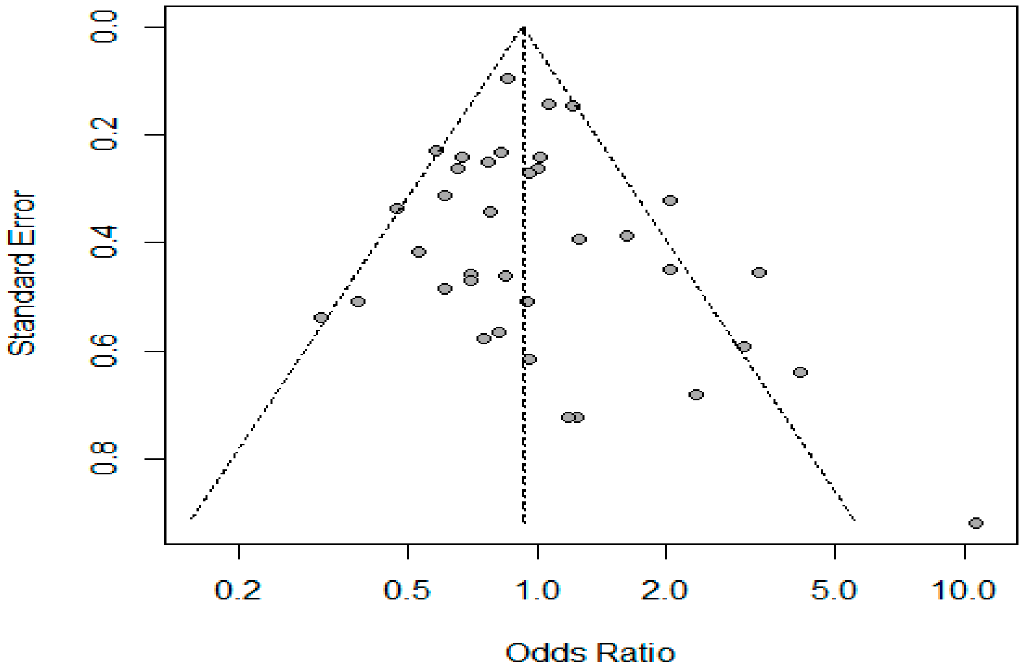

Figure S45. Funnel plot of the association between diabetes and sarcopenia.

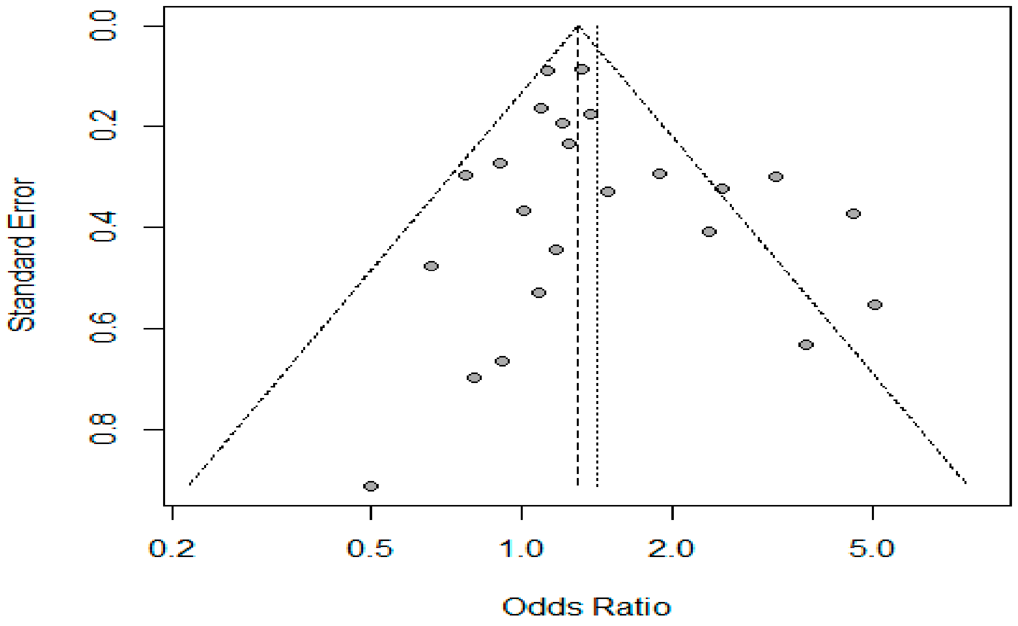

Supplement: Supplementary file 1 [file nutrients-13-04291-s001.zip › nutrients-1418872-supplementary.pdf]
